# A systematic review of hepatitis B virus (HBV) drug and vaccine escape mutations in Africa: A call for urgent action

**DOI:** 10.1371/journal.pntd.0006629

**Published:** 2018-08-06

**Authors:** Jolynne Mokaya, Anna L. McNaughton, Martin J. Hadley, Apostolos Beloukas, Anna-Maria Geretti, Dominique Goedhals, Philippa C. Matthews

**Affiliations:** 1 Nuffield Department of Medicine, University of Oxford, Oxford, United Kingdom; 2 Oxford University Academic IT Department, Oxford, United Kingdom; 3 Institute of Infection and Global Health, University of Liverpool, Liverpool, United Kingdom; 4 Division of Virology, University of the Free State/National Health Laboratory Service, Bloemfontein, Republic of South Africa; 5 Department of Microbiology and Infectious Diseases, Oxford University Hospitals NHS Foundation Trust, John Radcliffe Hospital, Headington, Oxford, United Kingdom; University of Geneva Hospitals, SWITZERLAND

## Abstract

International sustainable development goals for the elimination of viral hepatitis as a public health problem by 2030 highlight the pressing need to optimize strategies for prevention, diagnosis and treatment. Selected or transmitted resistance associated mutations (RAMs) and vaccine escape mutations (VEMs) in hepatitis B virus (HBV) may reduce the success of existing treatment and prevention strategies. These issues are particularly pertinent for many settings in Africa where there is high HBV prevalence and co-endemic HIV infection, but lack of robust epidemiological data and limited education, diagnostics and clinical care. The prevalence, distribution and impact of RAMs and VEMs in these populations are neglected in the current literature. We therefore set out to assimilate data for sub-Saharan Africa through a systematic literature review and analysis of published sequence data, and present these in an on-line database (https://livedataoxford.shinyapps.io/1510659619-3Xkoe2NKkKJ7Drg/). The majority of the data were from HIV/HBV coinfected cohorts. The commonest RAM was rtM204I/V, either alone or in combination with associated mutations, and identified in both reportedly treatment-naïve and treatment-experienced adults. We also identified the suite of mutations rtM204V/I + rtL180M + rtV173L, that has been associated with vaccine escape, in over 1/3 of cohorts. Although tenofovir has a high genetic barrier to resistance, it is of concern that emerging data suggest polymorphisms that may be associated with resistance, although the precise clinical impact of these is unknown. Overall, there is an urgent need for improved diagnostic screening, enhanced laboratory assessment of HBV before and during therapy, and sustained roll out of tenofovir in preference to lamivudine alone. Further data are needed in order to inform population and individual approaches to HBV diagnosis, monitoring and therapy in these highly vulnerable settings.

## Author summary

The Global Hepatitis Health Sector Strategy is aiming for the elimination of viral hepatitis as a public health threat by 2030. However, mutations associated with drug resistance and vaccine escape may reduce the success of existing treatment and prevention strategies. In the current literature, the prevalence, distribution and impact of hepatitis B virus (HBV) mutations in many settings in Africa are neglected, despite the high prevalence of HBV and co-endemic HIV infection. This systematic review describes the frequency, prevalence and co-occurrence of mutations associated with HBV drug resistance and vaccine escape mutations in Africa. The findings suggest a high prevalence of these mutations in some populations in sub-Saharan Africa. Scarce resources have contributed to the lack of HBV diagnostic screening, inconsistent supply of drugs, and poor access to clinical monitoring, all of which contribute to drug and vaccine resistance. Sustainable long-term investment is required to expand consistent drug and vaccine supply, to provide screening to diagnose infection and to detect drug resistance, and to provide appropriate targeted clinical monitoring for treated patients.

## Introduction

In 2015, the World Health Organisation (WHO) estimated that 3.5% of the world’s population (257 million people) were living with Hepatitis B virus (HBV) infection, resulting in 887,000 deaths each year, mostly from complications including cirrhosis and hepatocellular carcinoma (HCC) [1]. United Nations Sustainable Development Goals set out the challenge of elimination of viral hepatitis as a public health threat by the year 2030 [2]. One of the existing strategies in the elimination toolbox is use of antiviral drugs in the form of nucleos(t)ide analogues (NAs). Suppression of viraemia not only reduces inflammatory and fibrotic liver disease in the individual receiving treatment but also reduces the risk of transmission. However, the emergence of HBV resistance-associated mutations (RAMs) is a potentially significant concern for the success of this strategy.

Africa is the continent with the second largest number of individuals with chronic HBV (CHB) infection, with an estimated 6.1% of the adult population infected [1]. However, there is little commitment and resource invested into the burden of this disease, and many barriers are contributing to the epidemic [3,4]. Globally, less than 10% of the population with CHB are diagnosed, with an even smaller proportion on treatment [1,4]. This proportion is likely to be even lower in Africa. The situation in Africa is further complicated by the substantial public health challenge of coendemic human immunodeficiency virus (HIV) and HBV; coinfection worsens the prognosis in dually infected individuals [5]. There is also a lack of robust epidemiological data on HBV from Africa [3,4].

Widespread use of antiretroviral therapy (ART) for HIV, incorporating NAs that also have activity against HBV, may have an impact on HBV through improved rates of viraemic suppression, but also potentially by driving the selection of RAMs. The WHO recommends screening for Hepatitis B virus surface antigen (HBsAg) in all HIV-1 infected individuals prior to ART initiation, and for all pregnant women during antenatal visits, to improve the clinical outcomes of people living with CHB and to enhance interventions that reduce the incidence of new cases [6]. However, screening of HBsAg is not routinely performed in many settings in Africa, with lack of implementation at least partially driven by cost and lack of programmes for HBV treatment outside the setting of HIV coinfection. HBV infected patients either remain untreated (most typical in the setting of monoinfection), or are exposed to antiviral drugs without proper monitoring and often intermittently, putting them at risk of developing RAMs (more likely in the setting of HIV coinfection) [4,7–10].

**Fig 1:**
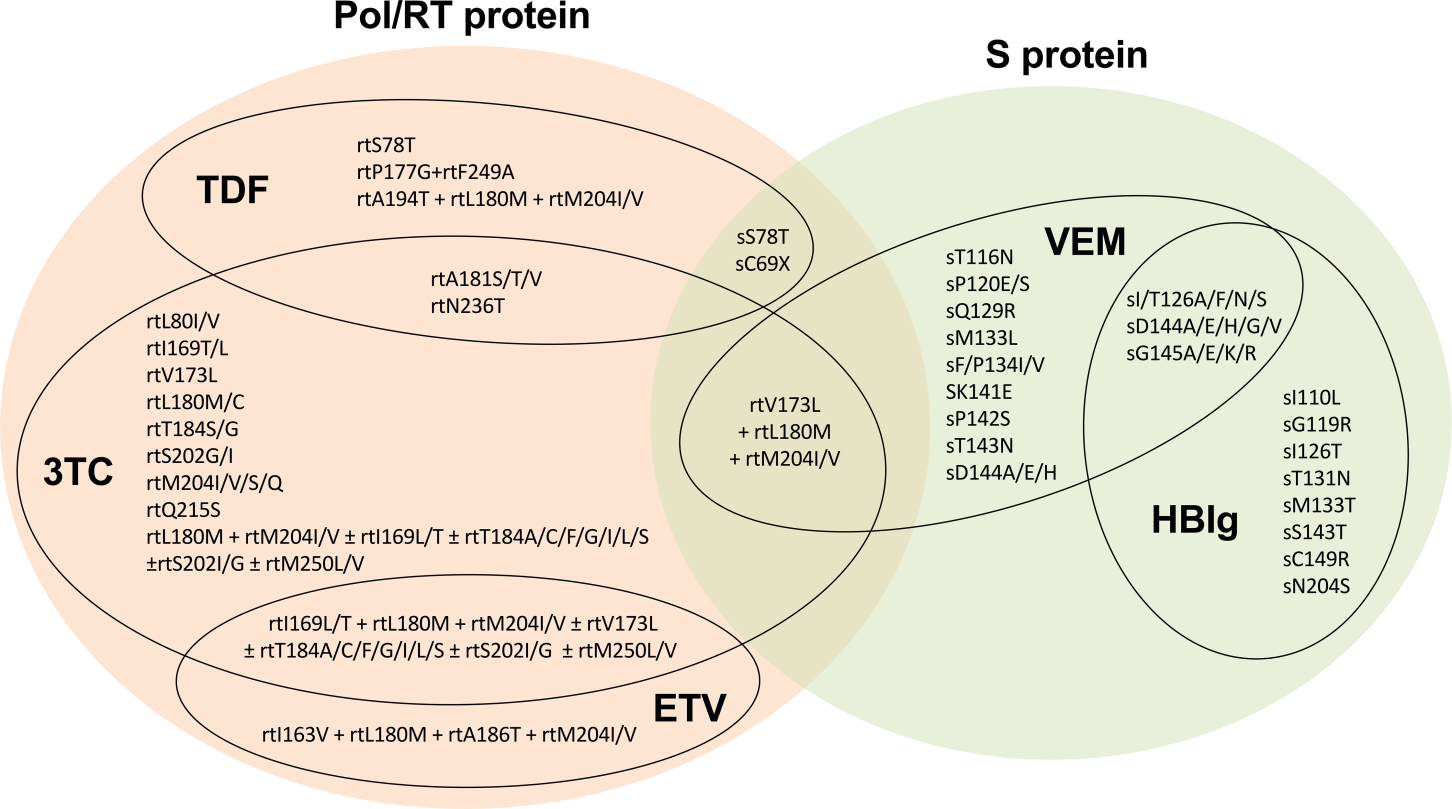
HBV drug resistance associated mutations (RAMs), vaccine escape mutations (VEMs) and mutations associated with Hepatitis B immunoglobulin (HBIg) resistance. HBV genes are shown in the coloured ovals. TDF = tenofovir, ETV = entecavir, 3TC = lamivudine. This figure incorporates data from eight studies; three were identified by the systematic review presented in this manuscript [12–14] and five from the wider literature [7,15–18].

HBV is a DNA virus that replicates via an RNA intermediate, with reverse transcriptase (RT) catalysing the transcription of RNA into DNA [7]. NAs that inhibit RT are therefore used to prevent HBV replication, including lamivudine (3TC), entecavir (ETV) and tenofovir (conventionally in the form of tenofovir disoproxil fumarate (TDF), but more recently available as the prodrug, tenofovir alafenamide fumarate (TAF)), with mostly historical use of other agents including telbivudine (LdT) and adefovir (ADV) [6,11]. Choice of TDF/TAF or ETV is determined by availability, cost, safety profile and barrier to resistance [4]. In Africa, the choice of agent is usually limited to 3TC or TDF. Emergence of mutations happens because the RT enzyme is error-prone and lacks the proofreading function required to repair errors during transcription [7]; when these mutations confer a selective advantage by allowing the virus to escape the effect of drug therapy, they will become amplified in the viral population. Some RAMs confer resistance to one agent only, while others are associated with resistance to several agents (Fig 1).

3TC was originally seen as a major breakthrough in treating HBV [19]. However, it is now known to have a low genetic barrier to resistance and its long-term effectiveness is limited as a result of resistance mutations in the ‘YMDD’ motif (tyrosine, methionine, aspartate, aspartate; amino acids 203–206) in domain C of the viral polymerase (Pol). These occur with associated upstream mutations in Pol domains A, B and in the B-C interdomain [7,15,16]. Among chronic HBV monoinfected patients, incidence of HBV resistance to 3TC is as high as 20% per year. In HIV/HBV coinfected patients, this can reach 90% over 5 years of treatment, as development of resistance is accelerated in HIV coinfection [5,20]. 3TC has also been associated with the induction of cross-resistance to emtricitabine (FTC), LdT, and at least partially ETV, thus reducing the options for subsequent treatment [10].

TDF is widely used in treatment of both HIV and HBV and is generally well tolerated. TDF has a high genetic barrier to resistance and maintains effective suppression of HBV in both monoinfected and HIV/HBV coinfected individuals [5,7,10,21,22]. Although it has a recognised association with nephrotoxicity in HIV treatment, current literature suggests it may be better tolerated in HBV infection [11]. Conversely, African populations have a higher background of renal disease [23] and could be potentially more vulnerable to nephrotoxitiy from TDF [24]. TAF delivers equally potent viraemic suppression at lower plasma levels, and is therefore associated with reduced nephrotoxicity [25], but is not available in Africa at present. HBV resistance to TDF is not well characterised, but there are emerging data from *in vitro* studies associating Pol mutations rtA194T and rtN236T with decreased susceptibility [11,21]. Virological breakthrough on TDF therapy has been reported in two patients harbouring rtS78T/sC69 mutations [17], and in another patient with multi-site polymerase mutations; rtL80M, rtL180M, rtM204V/I, rtA200V, rtF221Y, rtS223A, rtT184A/L, rtR153Q, and rtV191I [26]. The significance of these mutations needs to be further explored in clinical studies.

First line ART treatment regimens for HIV in sSA now almost universally include TDF, and current guidelines also recommend TDF-based regimens in individuals with HBV/HIV coinfection [27]. Accordingly, in both HIV monoinfection and HBV/HIV coinfection, use of TDF has increased across much of Africa. Nevertheless, it remains the case that 3TC is used as the only HBV-active agent in some settings [7,8], as well as in second line regimens, exemplified by South Africa where second line ART substitutes Zidovudine (AZT) for TDF leaving only 3TC coverage for HBV [28]. Among HBV/HIV coinfected children in South Africa treated with regimens including 3TC and/or TDF, HBV viraemia has been demonstrated, highlighting potential underlying HBV drug resistance [29].

ETV is another active agent, and is safe and well tolerated. However it is not active against HIV and therefore has to be added to ART regimens rather than being part of the primary backbone, is not recommended in pregnancy, and is not routinely available in most African settings [30]. Resistance arises more commonly in the context of prior 3TC exposure [11,31], which may limit its future potential in Africa, particularly in HIV endemic populations.

As a component of the Expanded Programme on Immunization (EPI), HBV preventive vaccines have been rolled out in Africa since 1995 [4]. HBV vaccine is highly effective in prevention of mother to child transmission (PMTCT); when administered to infants within 24 hours of birth followed by a dose given at 6 and another at 14 weeks to complete the primary series, it reduces the rate of mother to child transmission by 85%–95% [32,33]. However, by 2016 only 11 countries in Africa had adopted birth dose HBV vaccination as part of the routine infant immunisation schedule [34]. Changes in the S protein can result in vaccine escape mutants (VEMs) [16,18], and also diagnostic escape mutations which result in false negative HBsAg testing [16]. Mutations in HBV Pol can also lead to amino acid changes in the Surface (S) protein due to overlapping reading frames (ORFs) in the genome [16]. Whilst the S protein mutation sG145R has been identified as the major VEM, recently other mutations in S protein have been associated with immune escape [16] Fig 1. There are very few data for VEMs in Africa, but in other settings of high endemicity, VEMs can be common, as evidenced by a reported prevalence of 28% in vaccinated HBV-infected children in Taiwan [35].

To date, no systematic review has assessed the geography and prevalence of HBV RAMs and VEMs in Africa. An understanding of the extent to which these mutations circulate in Africa is essential to improving HBV therapy in patients with and without HIV coinfection. We therefore set out to describe the frequency, co-occurrence and distribution of RAMs and VEMs in Africa, and to suggest whether changes are needed in recommendations for laboratory diagnostics and/or approaches to drug therapy or vaccine deployment. This will underpin further research to identify and track relevant mutations in these populations.

## Methods

### Search strategy

Between October 2017 and January 2018, we searched the published literature, in MEDLINE (PubMed; https://www.ncbi.nlm.nih.gov/pubmed), SCOPUS (https://www.elsevier.com/solutions/scopus) and EMBASE (https://www.elsevier.com/en-gb/solutions/embase-biomedical-research). Our search strategy is detailed in S1 Table (documenting use of PRISMA criteria and selection of studies) and S2 Table (listing our search criteria). The earliest paper we identified on HBV drug resistance in Africa was published in 2007. We reviewed the titles and abstracts matching the search terms and only included those relating to drug or vaccine resistance in HBV infection, including only those that presented original data and had undergone peer review. All retrieved articles were in English, therefore no exclusion in relation to language was required.

For each publication we recorded reference, publication year, study design, sample size, study population, proportion of participants who tested HBsAg+ or HBV DNA+, country, year(s) of specimen collection, genotype identified, antiviral treatment, sequencing method, gene sequenced, number of sequenced samples, participant recruitment site and sequence accession number. Data were curated using MS Excel software (Microsoft, Redmond, WA).

### RAMs reported in published sequences not represented in primary studies

We expanded our search for evidence of RAMs by identifying publicly available HBV sequences from Africa, that had not been included in the results of our primary literature search. We used both the Hepatitis B Virus database (https://hbvdb.ibcp.fr/ [36] and Hepatitis Virus Diversity Research Alignments database (http://hvdr.bioinf.wits.ac.za/alignments/) [37].

### Analysis

In order to determine the prevalence of RAMs and VEMs, we first reported these using the denominator (total number of HBV positive patients) and numerator (total number of HBV positive patients with the specified mutation) as reported in published studies. We also pooled data by country in order to provide regional estimates. Downloaded sequences were managed using Sequence editor, database and analysis platform, SSE version 1.3, for analysis [38].

### Data visualisation

We developed an R package, gene.alignment.tables, for the visualisation of the sequence data in this study; this is available on Github [39] and can be used for visualising generic gene sequence datasets. The package was developed by University of Oxford’s Interactive Data Network and a specific instance of the visualisation is hosted as a Shiny app which can be viewed here: https://livedataoxford.shinyapps.io/1510659619-3Xkoe2NKkKJ7Drg/ [40].

## Results

The initial search yielded 56 articles in MEDLINE, 150 in SCOPUS and 150 in EMBASE. Of these, 32, 136 and 119 were excluded from search results of MEDLINE, SCOPUS and EMBASE respectively, as they did not did not meet the inclusion criteria. After de-duplication, 37 articles were included. 27 articles identified from MEDLINE, SCOPUS and EMBASE were identical; five unique articles were included from EMBASE, four from SCOPUS and one from MEDLINE. A total of 37 articles were downloaded in full (S1 Table (part II); S3 Table).

### Study characteristics

Epidemiological data for HBV represented by the 37 studies we identified are summarised in Table 1. Studies included were from Southern Africa (Botswana, Mozambique, South Africa, Zambia and Zimbabwe), East Africa (Ethiopia, Kenya, Malawi, Sudan and Uganda), West Africa (Cote d’Ivoire, Gambia, Ghana, Guinea-Bissau and Nigeria) and Central Africa (Cameroon, Gabon). There was considerable heterogeneity in recruitment protocols and exposure to anti-viral treatment. Twenty-six studies recruited from hospitals, three studies recruited from the community [8,41,42] and eight studies did not specify where recruitment was undertaken [10,43–49]. All studies were observational.

**Table 1:** Prevalence of HBsAg and HBeAg from 37 studies of HBV drug resistance in Africa.

	Study (Reference)	Country	Characteristics of study population	HIV co-infection status of cohort^a^	HBV prevalence in this cohort (reported as HBsAg prevalence unless otherwise specified)	HBeAg prevalence among HBV- positive individuals
**East Africa**	Deressa et al 2017 [50]	Ethiopia	Patients attending outpatient ART clinic at tertiary referral university hospital	+	17/308 (6%)	Not reported
	Hundie et al 2016 [41]	Ethiopia	Stored plasma samples from HBV infected blood donors obtained from blood bank centres	±	391/391 (100%)	Not reported
	Day et al 2013 [8]	Kenya	Longitudinal cohort study of female sex workers in an urban setting	+	11/159 (7%)	6/11 (55%)
	Kim et al 2011 [9]	Kenya	Individuals from an urban centre enrolled in randomised controlled trial of adherence to ART	+	27/389 (7%)	24/27 (89%)
	Mabeya et al 2017 [13]	Kenya	Individuals seeking treatment at the comprehensive HIV Clinic at tertiary referral university hospital	+	29/400 (7%)	Not reported
	Auodjane et al 2014 [20]	Malawi	Individuals starting ART treatment at a tertiary referral university hospital	+	133/1117 (12%)	67/133 (50%)
	Galluzzo et al 2012 [45]	Malawi	Pregnant women enrolled in a PMTCT study on safety and pharmacokinetics of antiretroviral drugs	+	21/21 (100%)	7/21 (33%)
	Mahgoub et al 2011 [42]	Sudan	Plasma samples from blood donors from capital city in Sudan	±	16/404 (4%)	Not reported
	Yousif et al 2014 [47]	Sudan	Individuals seeking treatment at a AIDS care unit and HIV treatment centre	+	96/358 (27%)^b^	32/ 50 (64%)
	Calisti et al 2015 [51]	Uganda	All HIV patients attending a regional referral hospital	+	109/2820 (4%)	Not reported
**West Africa**	Boyd et al 2015 [52]	Cote d’Ivoire	Individuals enrolled in randomised multi centre trials of benefits and risks of early ART initiation	+	259/ 2465 (11%)	39/168 (23%)
	Archampong et al 2017 [12]	Ghana	Serum samples from HBV-HIV co- infected patients collected at tertiary referral university hospital	+	235/235 (100%)	Not reported
	Chadwick et al 2012 [53]^d^	Ghana	Stored sera from all adult patients attending the HIV clinic at a tertiary referral university hospital	+	143/371 (39%)	Not reported
	Geretti et al 2010 [54]^d^	Ghana	Consecutive serum samples collected from unselected HIV-infected patients attending a tertiary referral university hospital	+	140/838 (17%)	37/140 (26%)
	Ndow et al 2017 [55]	Gambia	Individuals attending HIV clinic	+	23/187 (12%)	Not reported
	Stewart et al 2011 (44)	Gambia	Individuals receiving HAART; recruitment site not specified	+	70/ 570 (12%)	6/21 (29%)
	Langhoff Honge et al 2014 [56]	Guinea Bissau	Patients attending outpatient ART clinic at tertiary referral university hospital	+	94/576 (16%)	16/94 (17%), HDV prevalence: 18/72 (25%)
	Faleye et al 2015 [57]	Nigeria	Pregnant women attending antenatal clinics from two tertiary university hospitals	±	15/272 (6%)	Not reported
**Central Africa**	Gachara et al 2017 [58]	Cameroon	Patients attending outpatient ART health centre	+	20/337 (6%)^c^	Not reported
	Kouanfack et al 2012 [59]	Cameroon	Patients attending outpatient ART clinic at tertiary hospitals	+	54/552 (10%)	Not reported
	Magoro et al 2016 [60]	Cameroon	Patients attending outpatient ART health centre	+	116/445 (26%)	16/102 (16%)
	Bivigou-Mboumba et al 2016 [61]	Gabon	Patients attending outpatient ART clinic	+	71/762 (9%)	Not reported
	Bivigou-Mboumba et al 2018 [62]	Gabon	Patients attending HIV care centers	+	43/487 (9%)	Not reported
**Southern Africa**	Anderson et al 2015 [48]	Botswana	Stored plasma samples of HIV/HBV co-infected individuals collected from studies conducted in a Research Institution	+	81/81 (100%)	Not reported
	Matthews et al 2015 [63]	Botswana	Women attending antenatal and paediatric clinics	±	17/443 (4%)	16/60 (27%); HDV: negative
	Chambal et al 2017 [64]	Mozambique	Patients attending outpatient ART health centre	+	47/518 (9%)	Not reported
	Wandeler et al 2016 [14]	Mozambique	Individuals starting ART treatment at urban clinic in Mozambique and rural clinic in Zambia	+	78/1032 (8%)	24/168 (14%)
	Andersson et al 2013 [65]	South Africa	Stored serum of women infected with HIV enrolled in an Antenatal Sentinel HIV and Syphilis Prevalence Survey	±	97/3089 (3%)	17/94 (18%); HDV: negative
	Amponsah-Dacosta et al 2015 [43]	South Africa	Stored serum of individuals exposed to HBV participating in a health facility-based hepatitis B serosurvey conducted at a provincial level.	±	33/201 (16%)	Not reported
	Amponsah-Dacosta et al 2016 [49]	South Africa	Individuals due to HAART initiation enrolled in longitudinal study	+	5/5 (100%)	5/5 (100%)
	Hamers et al 2013 [10]	South Africa	Individuals enrolled in a multicentre prospective study of ART resistance monitoring	+	37/175 (21%)	Not reported
	Gededzha et al 2016 [66]	South Africa	Stored sera from HBV infected individuals attending a tertiary referral university hospital	±	8/9 (89%)	Not reported
	Makondo et al 2012 [46]	South Africa	Stored sera from HIV infected individuals prior to ART initiation, recruitment site not specified	+	71/298 (24%)^b^	Not reported
	Matthews et al 2015 [63]	South Africa	Women attending antenatal and paediatric clinics in South Africa and Botswana	±	49/507 (10%)	Not reported; HDV: negative
	Powell et al 2015 [67]	South Africa	Stored serum samples of individuals infected with HIV receiving care at a tertiary university hospital	+	37/394 (9%)	Not reported
	Selabe et al 2007 [68]	South Africa	Individuals infected with HBV admitted at tertiary University hospital	±	35/35 (100%)	Not reported
	Selabe et al 2009 [69]	South Africa	Individuals infected with HBV admitted at tertiary University hospital	-	17/17 (100)	9/17 (53%)
	Wandeler et al 2016 [14]	Zambia	Individuals starting ART treatment at urban clinic in Mozambique and rural clinic in Zambia	+	90/797 (11%)	24/168 (14%)
	Hamers et al 2013 [10]	Zambia	Individuals enrolled in a multicentre prospective study of ART resistance monitoring	+	55/523 (11%)	Not reported
	Baudi et al 2017 [70]	Zimbabwe	Stored plasma samples of individuals attending HIV support clinic	+	19/176 (11%)	Not reported

HBsAg and HBeAg prevalence were determined from 37 studies (Treatment naïve: n = 8 studies, 566 individuals with HBsAg; Treatment experienced: n = 19 studies, 1243 individuals with HBsAg; Mixed regimen where some were treatment experienced, naïve or treatment status not specified: n = 10 studies, 1046 individuals with HBsAg). Studies were identified by a systematic literature search of HBV resistance associated mutations (RAMs) and vaccine escape mutations (VEMs) from African cohorts published between 2007 and 2017 (inclusive). Note that three studies each appear twice in this table due to recruitment from more than one setting [10, 14, 63].

^a^ HIV status is designated ‘+’ whole cohort HIV-positive; ‘±’ some of cohort HIV-positive; ‘-’none of cohort HIV-positive

^b^ HBV prevalence in these cohorts was reported using HBV DNA detection rather than HBsAg

^c^ Occult HBV prevalence reported in these cohorts

^d^ These two studies recruited from the same overall cohort in Ghana.

Study populations were categorised as follows:

- HBV/HIV coinfected patients: (n = 28 studies), [8–10,12–14,20,43–48,50–56,58–62,64,67,70];
- HBV infected with and without HIV coinfection: (n = 8 studies), [41–43,57,63,65,66,68];
- Chronic HBV monoinfection: (n = 1 study), [69].

Antiviral treatment exposure varied as follows:

- Treatment-naïve: (n = 8 studies), [14,46–48,63,64,68,70];
- 3TC-based regimen only: (n = 10 studies), [8,9,20,44,45,49,53,59,61,69];
- Regimens including 3TC or TDF: (n = 6 studies), [10,13,51,52,55,65];
- Mixed regimen where some received 3TC, others TDF, while others left untreated; (n = 7 studies), [12,50,54,56,60,62,66];
- Treatment regimen not specified: (n = 6 studies), [41–43,57,58,67].

HBV amino acid polymorphisms were studied from within the following proteins;

- Pol only (n = 13 studies), [8,9,12–14,20,44,53,55,56,59,63,68]; only one of these used a deep sequencing method [20];
- S only (n = 3 studies), [43,57,65];
- Pol and S (n = 12 studies),[10,41,45,48,51,52,54,58,60,62,64,67];
- Pol, S and PC/BCP (n = 4 studies), [47,50,61,69];
- S and PC/BCP region (n = 3 studies), [42,46,70];
- Whole genome (n = 2 studies), [49,66].

All studies, except for two [53,68], specified the HBV genotype (S3 Table & S2 Fig).

### Prevalence of HBsAg, HBeAg and HDV coinfection

The prevalence rates of HBsAg in these study cohorts ranged from 3%-26%; however, the populations included were highly selected and therefore not necessarily representative of the general population, particularly as a result of a strong bias towards HIV-infection (Table 1). Only three studies included in this review reported on HDV prevalence: two studies did not detect any HDV antibodies [63,65], whereas the other study reported a HDV prevalence of 25% in Guinea-Bissau [56].

### RAMs identified in African cohorts

**Fig 2:**
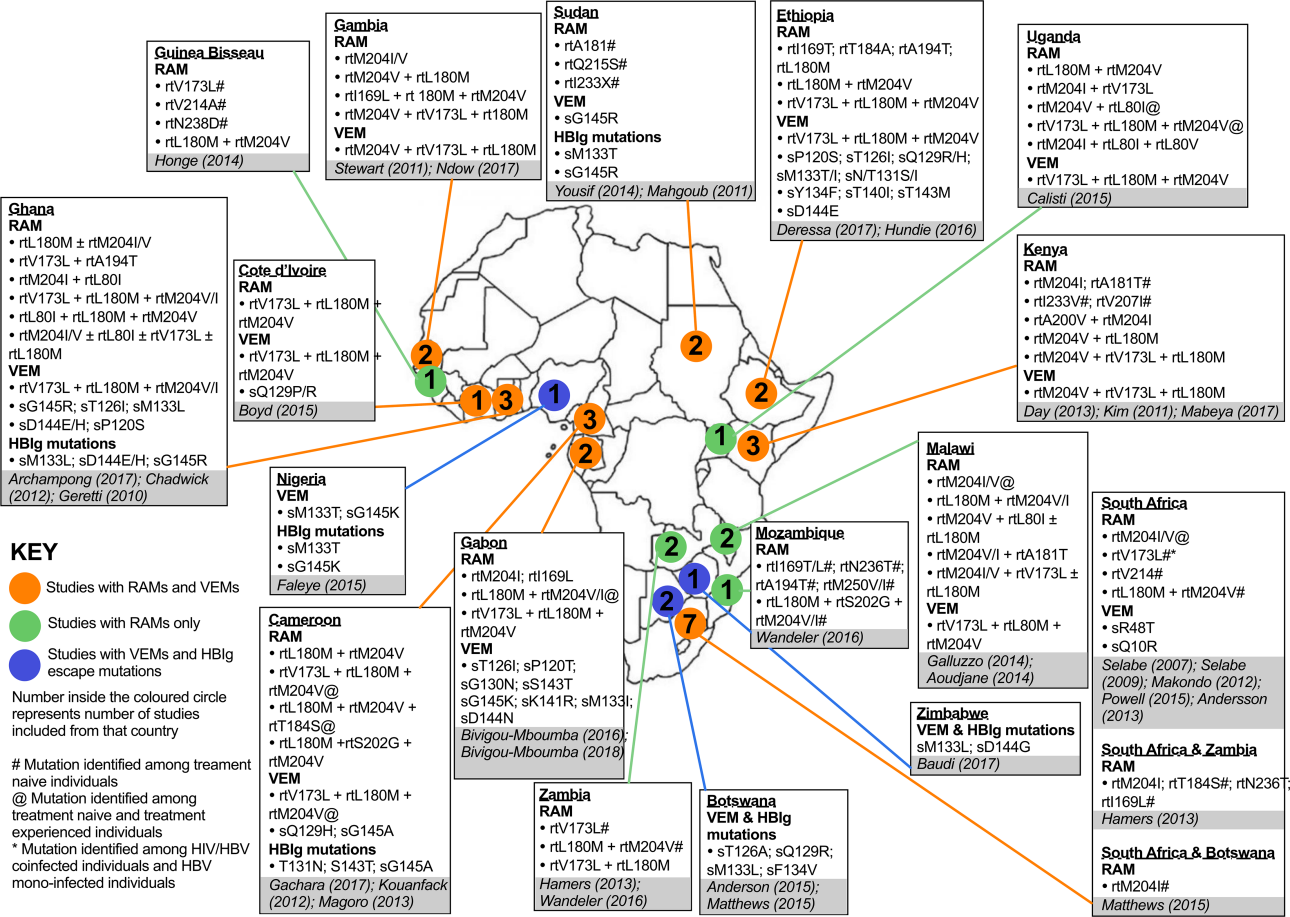
Annotated map to summarise HBV drug resistance associated mutations (RAMs) and vaccine escape mutations (VEMs). Mutations identified from 33 studies of African cohorts published between 2007 and 2017 (inclusive). Four studies identified by our systematic literature review were not represented here as they did not report any RAMs. Full details of each citation can be found in Table 1.

The co-occurrence and distribution of HBV RAMs and VEMs are summarised according to the region where they were identified (Fig 2). This illustrates the patchy and limited data that are available, with South Africa, Ghana and Cameroon best represented, but with large areas (especially in northern and central Africa) not represented at all in the literature.

Although 35 studies specified the HBV genotype, it was only possible to group RAMs according to genotype in fourteen studies [8,9,13,14,44,46,47,50,51,56,60–62,69] (S1 Fig; S2 Fig). The remaining 21 studies generally reported the genotypes identified, but did not specifically state the genotype of HBV within which RAMs were identified.

We have developed an interactive tool to display the genomic positions of RAMs identified through our literature review alongside relevant metadata. This can be accessed on-line here: https://livedataoxford.shinyapps.io/1510659619-3Xkoe2NKkKJ7Drg/ [40].

**Fig 3:**
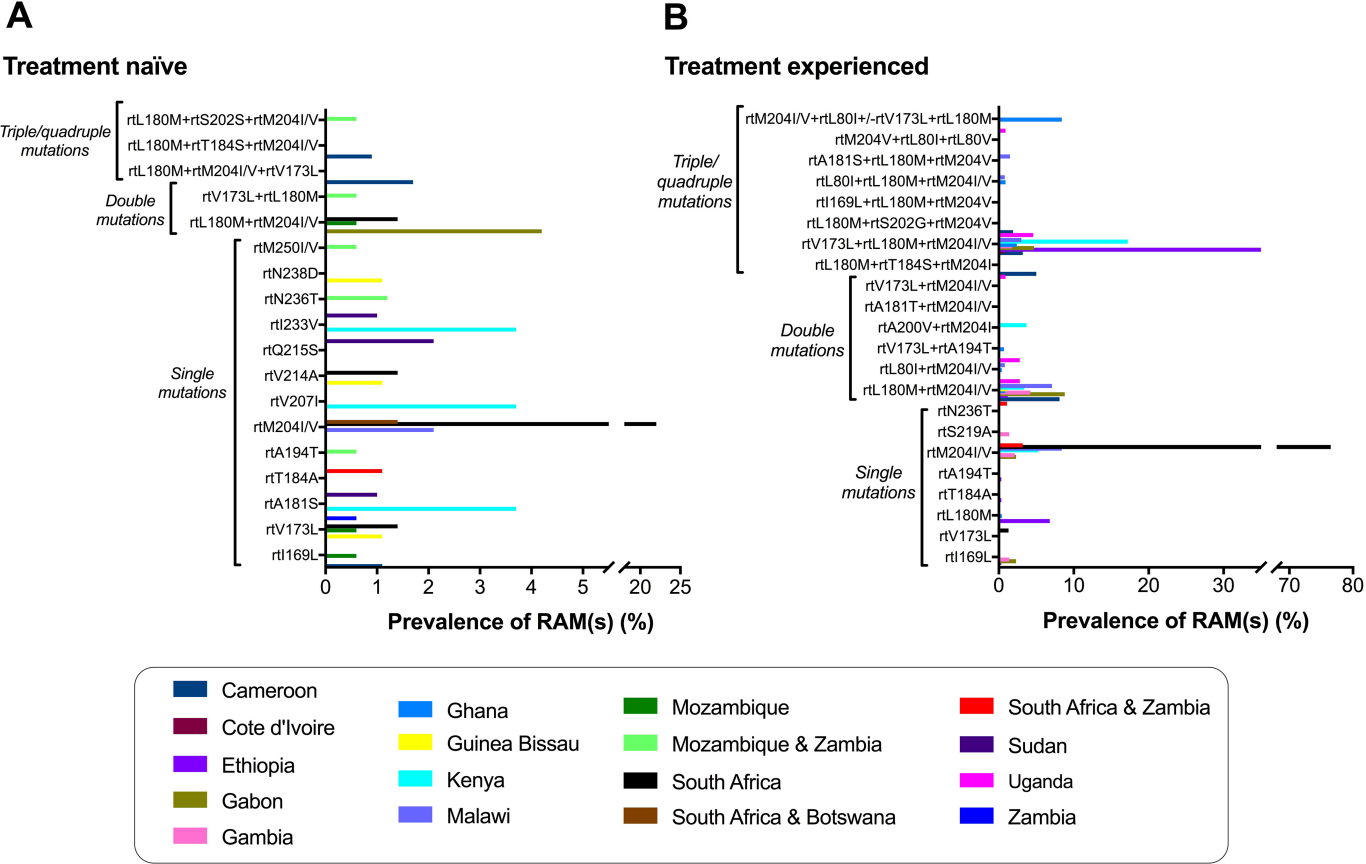
Prevalence of HBV resistance associated mutations (RAMs) in Pol/RT proteins among HBV infected patients in Africa. These data are derived from 27 studies of HBV drug resistance in Africa published between 2007 and 2017 (inclusive). The countries represented are listed in alphabetical order. A detailed summary of RAMs identified from each study is presented (Fig 2, S4 Table, S5 Table). Prevalence of RAMs for a specific country was determined by grouping all studies from that country that reported a specific mutation. We used all individuals who tested HBsAg positive to generate a denominator in order to provide a conservative estimate of RAM prevalence, and the numerator was the total number of individuals with that specific mutation from these studies. A: treatment naïve; B: treatment experienced.

Overall, the most prevalent RAM was rtM204V/I in both treatment experienced and treatment naïve individuals, and occurring either alone or in combination with other polymorphisms rtL80I/V, rtV173L, rtL180M, rtA181S, rtT184S, rtA200V and/or rtS202S (Fig 3); mutations among individuals with and without exposure to HBV therapy are listed in S4 Table and S5 Table, respectively). This mutation was present in 29 studies at a highly variable prevalence of between 0.4% [12] and 76% [69]. Across all cohorts, the mutation was present in 208/2569 (8%) of all individuals represented. The mutation, by itself, was most prevalent in South Africa; on pooling data for three studies from this setting, it was present in both treatment experienced and treatment naïve patients (n = 13/17, 76% [69] and n = 16/72, 22% [67,68] respectively). In addition to South Africa, rtM204I/V was also frequent in Malawi among treatment experienced patients (n = 24/154, 16% [20,45]) (Fig 3), and in genotype non-A infection: in this setting, the mutation was detected in genotype C infection (n = 2/17, 12% [69]) (S2 Fig).

The rtM204I/V mutation by itself confers resistance to 3TC; in combination with A194T it may also be associated with reduced efficacy to TDF, and in combination with L180M and V173L with vaccine escape, through corresponding substitutions in the surface antigen sites targeted by neutralising antibodies. Although TDF has a high genetic barrier to resistance, and is associated with reliable suppression of HBV viraemia [7,10,21,22], mutations rtN236T and rtA194T, which have been linked with resistance to both TDF and ADV [7], have been identified in Southern Africa in both treatment naïve [14] and treatment experienced [10] patients.

WHO guidelines recommend a first-line regimen including TDF in HIV/HBV coinfected patients [6], and the South African Department of Health HIV/AIDS treatment guideline included TDF as first-line regimen from 2010 [71], however we found a minority of studies (9/37, 24%) reporting TDF-containing regimens for HIV/HBV coinfected individuals. As anticipated, most of the studies that did use TDF were carried out after 2010, whereas those that used 3TC were generally earlier (S3 Table).

From this dataset, it is difficult to ascertain whether RAMs are genuinely more prevalent in genotype A infection, or this simply reflects enrichment of genotype A in sub-Saharan African populations (S2 Fig). Interpreting RAMs according to sub-genotypes was difficult since most studies did not specify sub-genotype and others did not indicate which RAMs were identified in which genotype. Of concern is the detection of RAMs even in reportedly treatment naïve individuals (Fig 3 & S4 Table), suggesting that RAMs are being transmitted. A study in South Africa that recruited 3TC-naïve HBV infected adults with or without HIV, reported rtM204I in 13/35 (37%) individuals [68].

### HBV RAMs in published sequences from Africa

We searched the Hepatitis B Virus database and GenBank to identify HBV sequences derived from Africa, from studies not already included in our review. We identified an additional 69 isolates: 23 had undergone full length genome sequencing whereas 46 isolates represented either the polymerase (n = 3) or S region (n = 43) of the HBV genome Table 2. To avoid duplication of results, we excluded fourteen studies already identified by our literature review that had submitted their sequences to GenBank (S3 Table). RAMs in the additional 69 isolates were as follows:

- rtM204V in genotype A (2/69, 2.9% of sequences), this occurred in combination with rtL180M;
- rtM204V + rtL180M in genotype E (1/69, 1.5%);
- rt180M + rtA181V in genotype E (1/69 (1.5%);
- rtQ215S identified in genotype D (4/69, 5.8%).

All these mutations are associated with 3TC resistance; rtA181V has also been associated with reduced susceptibility to TDF [7,15].

In the S gene, the most prevalent mutations were:

- sD144A/E/G occurring in genotype A (6/69, 8.7%), D (10/69, 14.5%) and E (7/69, 10.1%) associated with VEM;
- sI110L occurring in genotype A (3/69, 4.3%), D (4/69, 5.8%) and E (11/69, 15.9%) associated with immunoglobulin resistance.

**Table 2:** HBV drug resistant mutations (RAMs) identified from HBV genome sequences from Africa downloaded from the Hepatitis B Virus database (https://hbvdb.ibcp.fr/) [36] and GenBank database (http://hvdr.bioinf.wits.ac.za/alignments/) [37].

HBV Protein	HBV Genotype	Position and nature of the mutation	Number (%) of HBV sequences with mutation	Accession number	Region sequenced	Country of origin
Polymerase (Pol)	A	rtL180M	2/69 (2.9%)	KM519454	Full length	South Africa
				FM199980	Full length	Rwanda
		rtM204V	2/69 (2.9%)	KM519454	Full length	South Africa
				FM199980	Full length	Rwanda
		rtI233V	1/69 (1.4%)	HM535205	Full length	Zimbabwe
	D	rtV214A	1/69 (1.4%)	FJ904395	Polymerase	Tunisia
		rtQ215S	4/69 (5.8%)	FJ904414	Full length	Tunisia
				FJ904431	Full length	Tunisia
				FJ904436	Full length	Tunisia
				FJ904438	Full length	Tunisia
	E	rtV173L	1/69 (1.4%)	KF849723	Full length	Angola
		rtL180M	2/69 (2.9%)	KF849720	Full length	Angola
				KF849723	Full length	Angola
		rtA194T	1/69 (1.4%)	GQ161771	Full length	Guinea
		rtM204V	1/69 (1.4%)	KF849723	Full length	Angola
		rtA181V	1/69 (1.4%)	KF849720	Full length	Angola
		rtN238D	2/69 (2.9%)	HM363566	Polymerase	Nigeria
				HM363587	Polymerase	Nigeria
Surface (S)	A	sI110L	3/69 (4.3%)	KY493896	S	Cameroon
				KP168431	Full length	Kenya
				AY233286	Full length	South Africa
		sP120S	1/69 (1.4%)	KX648547	S	Zimbabwe
		sG129R	1/69 (1.4%)	FN547352	S	Cameroon
		sT126A	1/69 (1.4%)	JN182330	S	South Africa
		sD144A/E/G	6/69 (8.7%)	FN547249	S	Cameroon
				KX493873	S	Cameroon
				FM199980	Full length	Rwanda
				FM200180	S	Rwanda
				FM200189	S	Rwanda
				KF467020	S	South Africa
		sG145R	1/69 (1.4%)	FM200185	S	Rwanda
		sC149R	1/69 (1.4%)	KF476024	S	South Africa
	D	sI110L	4/69 (5.8%)	AB561830	S	Egypt
				KX357627	Full length	Ethiopia
				FJ904429	S	Tunisia
				KJ416196	S	Tunisia
		sP120S	1/69 (1.4%)	KX357636	Full length	Ethiopia
		sM133T	1/69 (1.4%)	KM108592	S	Sudan
		sD144E	10/69 (14.5%)	FJ904427	Full length	Tunisia
				FN547165	S	Cameroon
				FN547179	S	Cameroon
				FN547239	S	Cameroon
				FN547255	S	Cameroon
				FN547258	S	Cameroon
				FN547262	S	Cameroon
				FN547281	S	Cameroon
				FN547318	S	Cameroon
				FN547319	S	Cameroon
	E	sI110L	11/69 (15.9%)	KY494047	S	Cameroon
				AM494711	S	Central Africa Republic
				AM494720	S	Central Africa Republic
				AM494725	S	Central Africa Republic
				AM494727	S	Central Africa Republic
				JQ972822	S	Central Africa Republic
				AB205190	Full length	Ghana
				GQ161756	S	Guinea
				GQ161768	Full length	Guinea
				GQ161795		Guinea
				DQ060822	Full length	South Western Africa and Madagascar
		sT126N	1/69 (1.4%)	HM363608	S	Nigeria
		sP120S	1/69 (1.4%)	HM363599	Full length	Nigeria
		sM133T	3/69 (4.3%)	HM363603	S	Nigeria
				KF170751	S	Sudan
				KF170752	S	Sudan
		sD144E	7/69 (10.1%)	FN547300	S	Cameroon
				KY494047	S	Cameroon
				AM494719	S	Central African Republic
				AM494726	S	Central African Republic
				FN594756	Full length	Niger
				HM363565	Full length	Nigeria
				HM363590	S	Nigeria
		G145A/R	3/69 (4.3%)	KY493921	S	Cameroon
				AB205327	S	Ghana
				AM494741	S	Central African Republic

### VEMs

VEMs were identified in Central, East, West and Southern Africa (Fig 2). However, it was not possible to ascertain whether individuals harbouring these mutations had been vaccinated against HBV infection. The most common VEM was the triple mutation rtV173L + rtL180M + rtM204I/V, found in the *Pol* gene. This suite of mutations was identified in 14 studies [12,13,20,44,50–55,58–60,62], at a pooled prevalence of 4% (57/1462). Another significant VEM, sG145K/R [16], was identified in six studies [12,42,47,57,60,62] and sM133L/T, associated with VEM, immunoglobulin and diagnostic escape mutation [12,48], was identified in seven studies [12,41,47,48,57,62,70] (Fig 2).

## Discussion

### Summary

To our knowledge, this is the first systematic review that assesses RAMs and VEMs for HBV in Africa. The high rates of HBV infection among HIV infected individuals in some locations including Cameroon [60] and South Africa [10] could be an indication that HBV infection has been previously under-reported, possibly due to lack of routine screening, poor awareness, stigma, high costs and limited clinical and laboratory infrastructure [4,8–10,45,53]. The literature suggests a widespread exposure of the HIV-infected population to 3TC-based treatment. This may be changing over time in line with current ART treatment recommendations (regimens for Africa summarised in S6 Table), but the introduction of TDF-based regimens for HIV treatment has been inconsistent, and TDF monotherapy is not consistently available for HBV infection in the absence of HIV.

In keeping with other settings, the most common RAM identified here was rtM204I/V, either alone or in combination with compensatory mutations rtL180M ± rtV173L. Of concern, rtM204V/I was seen in 76% of treatment experienced patients [69] and 22% of treatment naïve patients [67,68] in South Africa. A review of worldwide incidence of RAMs among treatment naïve patients also described rtM204V/I as the most frequent, but with a much lower prevalence of 5% [72]. The contribution of unreported or undocumented 3TC exposure in the reportedly treatment naïve populations remains to be determined. A European study demonstrated that the most frequent primary mutation was rtM204V/I, found in 49% of treatment experienced patients [73], while in China rtM204I, rtN236T and rtL180M+rtM204V+rtV173L/rtS202G were also the most prevalent RAMs [74].

The triple mutation rtM204V + rtL180M + rtV173L has been identified in East, West and Central Africa [20,44,51–54,59]. This combination of polymorphisms is associated with both vaccine escape and resistance to 3TC and other L-nucleoside analogues [20,44,51,54,59,60]. Interestingly, this triple mutation has not been reported in the Southern African region to date, which is likely to reflect the composition of the study populations.

### Clinical and public health significance of RAMs

Apart from the nature of drugs being used for HBV treatment, other reported predictors of HBV drug resistance include HBV viral load, HBV intra host heterogeneity, HBeAg status, host body mass index and serum alanine aminotransferase (ALT) activity [20,75,76]. Individuals with rtM204V/I plus compensatory mutations typically exhibit high HBV DNA levels [20] and are therefore highly infectious to others. The spread of RAMs may lead to a rise in drug resistance in treatment naïve chronic HBV infection, representing a substantial challenge for Africa and highlighting an imperative to ensure routine use of TDF in preference to, or in combination with, 3TC-based therapy.

Although these data provide a preliminary picture of the prevalence of RAMs in some settings, there are no recommendations to stipulate any specific prevalence threshold above which HBV drug resistance mutations represent a significant barrier to successful treatment at a population level, and/or RAM prevalence thresholds that should trigger a switch to alternative first-line therapy. For HIV, surveillance for transmission of RAMs is based on screening recently infected, treatment naive individuals, and classifies drug resistance using thresholds of <5%, 5–15%, and >15% to stratify the risk to public health [77]. Similar thresholds and recommendations for HBV could help to underpin the assimilation of epidemiological data and to unify treatment approaches.

### TDF resistance

The identification of mutations associated with reduced TDF susceptibility are of concern, as they suggest the potential for increasing prevalence of polymorphisms that confer partial or complete viral escape from a drug that to date has not been widely associated with resistance. There is now potential for increasing selection of TDF resistance as this drug becomes more widely used. However, as a new first line single tablet option incorporating 3TC, TDF and Dolutegravir (DTG) (triple therapy abbreviated to ‘LTD’) emerges as a recommended option for HIV treatment in Africa, surveillance is needed to determine the clinical outcomes for HBV [78].

If clinically significant, TDF resistance mutations may still represent a particular problem for many African settings, as resource constraints make it unrealistic to provide baseline screening for RAMs, or to monitor patients on treatment with serial viral load measurements. Despite these potential concerns, it has been shown that TDF is effective even in the presence of RAMs and that there is comparable efficacy among 3TC-experienced and NA-naïve patients [79].

### VEM

VEM were identified in 16 different countries in East, West, Central and Southern Africa. Information on vaccine exposure was not available, but there are two strands of evidence to support significant population exposure to HBV vaccination. First, vaccination has been progressively rolled out in most countries in sSA since the mid-1990’s; second, most HBsAg mutations reported by these studies are located within the common immunodominant B cell epitope (aa 124–147) in which selection of polymorphisms is associated with HBV vaccination [80,81].

VEM have been more robustly reported from Asia, in settings where the HBV infant vaccination programme is well established; for example, in Taiwan, VEM prevalence among vaccinated children increased from 7.8% to 23.1% within 15 years of the launch of the universal vaccine program, although the decline in VEM prevalence thereafter may be partly related to a smaller HBV carrier pool [80]. HBV infection despite immunoprophylaxis can occur either as a consequence of MTCT of pre-existing VEM, or as a result of *de novo* selection of escape mutations from vaccine- induced immune responses, particularly in the setting of delayed vaccination [80,81].The HBV genotype sequence used for vaccines may potentially have an influence on immunogenicity against non-vaccine genotypes, but there are limited data to support this [82]. Only 11 African countries recommend the first HBV vaccine dose at birth, in contrast to the majority of African countries in which HBV vaccination is delayed until 6 weeks of age [33]. It is likely that this delay not only provides a window of infection but also increases the possibility of transmitted VEM and/or emergence of new escape mutations.

High maternal HBV viral load and immunosuppression are other risk factors associated with VEM among infants [80]; both of these are pertinent for emergence of VEMs in Africa given that HBV viral load testing is not routinely available, and HIV is highly prevalent in some populations. Effective PMTCT strategies in Africa, including screening and treating antenatal women, increasing access to viral load monitoring, and introducing HBV birth dose vaccine will help to decrease the prevalence of VEM [4,33,83].

### HDV/HBV coinfection

One study from our literature review reported a high HDV prevalence of 25%; however, in this cohort, RAMs occurred in individuals with HBV monoinfection [56]. Given that HDV is characteristically associated with decreased HBV replication [84], it is possible that emergence of HBV RAMs is altered in this setting. However, as the true prevalence and impact of HDV in sSA is not known [85], further studies are needed to determine the impact of HDV coinfection on HBV RAMs.

### Limitations of current data

Screening for HBV infection is not routinely performed in many African settings and therefore the true prevalence and characteristics of HBV infection are not known [4,7–10]. We identified very few published studies; only a minority of patients had HBV sequencing undertaken, and there were no data from certain regions of Africa. This highlights the substantial problem of HBV neglect in Africa, and a specific blind-spot relating to sequence data [4]. Identifying the true prevalence of resistance mutations, and characterising the populations in which these are selected and enriched, is currently not possible due to sparse data and lack of clear descriptions of the denominator population. Most such studies do not perform a truly systematic assessment, but focus on high risk groups–particularly including those with HIV/HBV coinfection: of the 37 studies included here, only one exclusively reported on participants who were HBV mono-infected [69]. Although we have made every effort to assimilate the relevant data to build up a regional picture for Africa, the heterogeneity between studies makes it difficult to draw robust conclusions from pooled data. These findings are a reflection of the little attention paid towards the burden of this disease in Africa and the neglect in robust epidemiological data.

Only nine studies undertook a longitudinal approach to detection of drug resistance [8–10,20,44,45,49,52,53]. The results of the other 28 studies that undertook a cross-sectional approach could be skewed by the timing of recruitment of study participants, with a risk of under-representation of drug resistance if screening is undertaken only at baseline, and potentially an over-representation if screening is undertaken in patients with HIV coinfection, who are more at risk of advanced disease and prolonged drug exposure. As most of these studies recruited individuals from hospital settings, this raises the latter possibility.

Mutations across the whole genome might be relevant in determining resistance [86]. However, most of the included studies analysed only defined genes from within the HBV genome; only two sequenced the whole genome, and these determined consensus sequence. This potentially results in an under-representation of RAMs and VEMs that may be present as low numbers of quasispecies, but could become significant if selected out by exposure to drug or vaccine.

In studies that reported RAMs among treatment naïve individuals, the literature suggests that sequence analysis was performed prior to ART initiation. However, we cannot exclude the possibility that that some of these participants had prior ART exposure. Due to the nature of the cohorts that have been studied, most of the RAMs identified were from HIV/HBV coinfected individuals. It is possible that HIV increases the risk of HBV RAMs both in terms of drug exposure, and also as a function of increased HBV viral loads. A study from Malawi demonstrated the rapid emergence of 3TC resistance in HIV coinfection, with virtually all treatment naïve HBeAg positive individuals starting antiviral treatment showing emergence of rtM204I by six months. Likewise, a study carried out in Italy revealed that patients with HIV coinfection were more likely to harbour the rtM204V mutation and to show multiple mutations compared to HBV monoinfected patients [87]. It would be worth further exploration of this observation in Africa, as there are currently very limited data.

### Challenges and opportunities for Africa

A major challenge for Africa is to improve coverage rates of infant vaccination, deploy catch-up vaccination programmes for older children and adults, adopt widespread screening and develop treatment programmes for HBV. While HBV vaccine is effective, gaps in vaccine coverage in Africa can be demonstrated by the high perinatal transmission rate of HBV in sSA (estimated at 38% among women with a high HBV viral load) and the observation that up to 1% of newborns in sSA are still infected with HBV [88]. Sustained efforts are required to build robust PMTCT programmes that deliver screening and treatment for antenatal women, and timely administration of HBV birth vaccine for their babies [33,83].

Although the WHO recommends monitoring for the development of drug resistance once on therapy [6], implementation remains challenging as viral load monitoring and sequencing are both rarely available [7]; despite the advancement and availability of HIV testing and monitoring, in many settings it remains uncommon to monitor HIV viral load after ART initiation [89,90]. Affordable, accessible and sustainable platforms for quantifying both HIV and HBV viral loads remain an important priority for many settings in Africa, given the lack of on-treatment monitoring in many settings. Given the simplicity and relative ease of collection, preparation and transport of dried-blood-spot (DBS) samples [91], adopting DBS testing could improve access to HBV diagnosis, viral load monitoring and linkage to care, especially in areas with limited access to laboratory facilities.

Development of a cheap, rapid test for the detection of the most frequently observed RAMs and VEMs should be considered as a potentially cost-effective strategy for Africa. Proof of principle for a rapid test for diagnosis and detection of resistance has been demonstrated by the GeneXpert MTB/RIF assay for *Mycobacterium tuberculosis* (MTB) [92]. A similar approach has been applied for HBV through use of a multiplex ligation-dependent probe real time PCR (MLP-RT-PCR) [93]. Although this assay is able to detect RAMs quickly and cheaply, there are still limitations as the test requires high viral load samples, is based on detection of known RAMs from within discrete regions of the genome, and may not identify RAMs that are present as minor quasispecies.

New metagenomic sequencing platforms, such as Illumina and Nanopore, provide the opportunity for whole deep genome sequencing, which can reveal the full landscape of HBV mutations in individual patients, quantify the prevalence of drug resistance mutations among HBV quasi-species, and determine the relationship between these polymorphisms and treatment outcomes [87]. Nanopore technology also has the potential to develop into an efficient point of care test that could detect viral infection and coinfection, as well as determining the presence of VEMs and RAMs [94], but is currently limited by cost and concerns about high error rates.

There have been few studies looking at the correlation between genotype, clinical outcomes of disease, response to antiviral therapy and RAMs/VEMs, but none from Africa. Studies outside Africa have shown that genotype A is more prone to immune/vaccine escape mutants, pre-S mutants associated with immune suppression, drug associated mutations and HCC in HIV/HBV coinfected participants [46,87,95]. Studies investigating the role of genotypes in predicting response to antiviral therapy and their association with various types of mutations are urgently needed in Africa, particularly in light of the high frequency of genotype A infection and high population exposure to antiviral agents that have been rolled out over the past two decades as a component of first-line ART.

Existing infrastructure for diagnosis, clinical monitoring and drug therapy for HIV represents an opportunity for linkage with HBV care. Particularly in settings of limited resource, joining up services for screening and management of blood-borne virus infection could be a cost-effective pathway to service improvements.

### Conclusions

This review highlights the very limited data for HBV RAMs and VEMs that are available from Africa. Scarce resources resulting in lack of diagnostic screening, inconsistent supply of HBV drugs and vaccines, and poor access to clinical monitoring contribute to drug and vaccine resistance, potentially amplifying the risk of ongoing transmission and adding to the long-term burden of HBV morbidity and mortality in Africa. We call for urgent action to gather and analyse better data, particularly representing the HBV monoinfected population, and for improved access to TDF.

HBV RAMs and VEMs have been identified in several African countries among HIV/HBV coinfected and HBV monoinfected patients, before and during treatment with NAs but the data are currently insufficient to allow us to form a clear picture of the prevalence, distribution or clinical significance of these mutations. Overall, the data we describe suggest a significantly higher prevalence of drug resistance in some African populations than has been described elsewhere, and that is not confined only to drug-exposed populations, highlighting an urgent need for better population screening, assessment of HBV infection before and during therapy, and increasing roll out of TDF in preference to 3TC. At present, TDF accessibility is largely confined to HIV/HBV coinfected individuals; we now need to advocate to make monotherapy available for HBV monoinfected individuals. However, there are uncertainties as to whether its long-term use might result in nephrotoxicity, and potentially in an increase in selection of TDF RAMs.

We should ideally aim for the goals of a combined HBV test that includes diagnosis of infection, genotype and presence of RAMs/VEMs; new sequencing platforms such as Nanopore make this technically possible, although cost remains a significant barrier at present. Sustainable long-term investment is required to expand consistent drug and vaccine supply, to provide screening infection and for drug resistance, and to provide appropriate targeted clinical monitoring for treated patients.

## Supporting information

S1 Fig HBV drug resistance associated mutations (RAMs) grouped according to genotype. Data summarised from fourteen studies published between 2009–2017 (inclusive). 21 studies were not represented here as they did not specifically indicate which genotype individuals with RAMs belonged to. **Available at** https://doi.org/10.6084/m9.figshare.5774091 [96].
(TIF)

S2 Fig Distribution of HBV genotypes and prevalence of HBV resistance associated mutations (RAMs) in Pol/RT proteins in geno-A and geno-non-A samples. A: Distribution of HBV genotypes derived from 35 studies reporting resistance associated mutations (RAMs) in Africa published between 2009 to 2017 (inclusive); B: Prevalence of HBV resistance associated mutations (RAMs) in Pol/RT proteins in geno-A and geno-non-A samples. These data are derived from 14 studies of HBV drug resistance in Africa published between 2007 and 2017 (inclusive). 21 studies were not represented here as they did not specifically indicate which genotype individuals with RAMs belonged to. We had more geno-A samples represented than other samples, we therefore combined samples from other genotypes that had RAMs (B, C, D, E, D/E) to form geno-non-A samples. We then compared prevalence of Pol/RT mutation between geno-A samples to geno-non-A samples. Prevalence of RT/Pol mutations for a specific genotype(geno-A/geno-non-A) was determined by grouping all studies with geno-A/geno-non-A infection that reported a specific mutation; the denominator was the total number of individuals infected with geno-A/geno-non-A from these studies and the numerator was the total number of individuals infected with geno-A/geno-non-A with that specific mutation. **Available at** https://doi.org/10.6084/m9.figshare.5774091 [96].
(TIF)

S1 Table PRISMA (preferred reporting items for systematic reviews and meta-analyses) criteria for a systematic review of hepatitis B virus (HBV) drug and vaccine escape mutations in Africa. Available at https://doi.org/10.6084/m9.figshare.5774091 [96].
I. Checklist to demonstrate how PRISMA criteria (2009) have been met in this review;
II. Flow diagram illustrating identification and inclusion of studies for a systematic review of drug and vaccine resistance mutations in Africa.
(PDF)

S2 Table Details of search strategy used to identify studies on HBV resistance associated mutations (RAMs) and vaccine escape mutations (VEMs) conducted in Africa. A: PubMed database; B: SCOPUS and EMBASE database. **Available at** https://doi.org/10.6084/m9.figshare.5774091 [96].
(PDF)

S3 Table Full details of 37 studies identified by a systematic literature search of HBV resistance associated mutations (RAMs) and vaccine escape mutations (VEMs) from African cohorts published between 2007 and 2017 (inclusive). **Available at** https://doi.org/10.6084/m9.figshare.5774091 [96].
(PDF)

S4 Table HBV Pol/RT mutations among treatment-naïve HBV infected patients in Africa from 12 studies published between 2007 and 2017 (inclusive). **Available at** https://doi.org/10.6084/m9.figshare.5774091 [96].
(PDF)

S5 Table HBV Pol/RT mutations among treatment-experienced HBV infected patients in Africa, from 25 studies published between 2009 and 2017 (inclusive). Available at https://doi.org/10.6084/m9.figshare.5774091 [96].
(PDF)

S6 Table First line ART regimen for adults in Africa, and overlap with HBV therapy. **Information derived from published ART guidelines in all cases where these are available in the public domain. This information was collated in May 2018.** Available at https://doi.org/10.6084/m9.figshare.5774091 [96].
(PDF)

## References

[1] World Health Organization. Global Hepatitis Programme. Global hepatitis report, 2017. http://www.who.int/hepatitis/publications/global-hepatitis-report2017/en/

[2] WHO Combating Hepatitis B and C to reach elimination by 2030. Advocacy brief. 2016. http://apps.who.int/iris/bitstream/10665/206453/1/WHO_HIV_2016.04_eng.pdf

[3] Lemoine M, Thursz MR. Battlefield against hepatitis B infection and HCC in Africa. J Hepatol. 2017 66: 645–654. 10.1016/j.jhep.2016.10.013 27771453

[4] O’Hara GA, McNaughton AL, Maponga T, Jooste P, Ocama P, Chilengi R, et al Hepatitis B virus infection as a neglected tropical disease. PLoS Negl Trop Dis. 2017 11:10. 10.1371/journal.pntd.0005842 PMC5628785 28981505

[5] Ocama P, Seremba E, Apica B, Opio K. Hepatitis B and HIV co-infection is still treated using lamivudine-only antiretroviral therapy combination in Uganda. Afr Health Sci. 2015 15(2):328–33. 10.4314/ahs.v15i2.4 26124776 PMC4480502

[6] WHO Hepatitis B treatment guidelines. 2015 http://www.who.int/mediacentre/news/releases/2015/hepatitis-b-guideline/en/

[7] Beloukas A, Geretti AM. Hepatitis B Virus Drug Resistance. In: Antimicrobial Drug Resistance; 2017p.1227–42.

[8] Day SL, Odem-davis K, Mandaliya KN, Jerome KR, Cook L, Masese LN, et al Prevalence, Clinical and Virologic Outcomes of Hepatitis B Virus Co-Infection in HIV-1 Positive Kenyan Women on Antiretroviral Therapy. PloS One. 2013 8(3):1–5. 10.1371/journal.pone.0059346 PMC3601052 23527168

[9] Kim HN, Scott J, Cent A, Cook L, Morrow RA, Richardson B, et al HBV lamivudine resistance among hepatitis B and HIV coinfected patients starting lamivudine, stavudine and nevirapine in Kenya. J Viral Hepat. 2011 18(10):447–52. 10.1111/j.1365-2893.2011.01466.x PMC3177102 21914062

[10] Hamers RL, Zaaijer HL, Wallis CL, Siwale M, Ive P, Botes ME, et al HIV-HBV coinfection in Southern Africa and the effect of lamivudine- versus tenofovir-containing cART on HBV outcomes. J Acquir Immune Defic Syndr. 2013 64(2):174–82. 10.1097/QAI.0b013e3182a60f7d 23892239

[11] Fung J, Lai C-L, Seto W-K, Yuen M-F. Nucleoside/nucleotide analogues in the treatment of chronic hepatitis B. J Antimicrob Chemother. 2011; 66(12):2715–25. 10.1093/jac/dkr388 21965435

[12] Archampong TNA, Boyce CL, Lartey M, Kwamena W, Obo-akwa A, Kenu E, et al HBV genotypes and drug resistance mutations in antiretroviral treatment-naïve and treatment-experienced HBV-HIV co-infected patients. Antivir Ther. 2017;22(1):13–20. 10.3851/IMP3055 27167598 PMC5106338

[13] Mabeya SN, Ngugi C, Lihana RW, Khamadi SA, Nyamache AK. Predominance of Hepatitis B Virus Genotype A Among Treated HIV Infected Patients Experiencing High Hepatitis B Virus Drug Resistance in Nairobi, Kenya. AIDS Res Hum Retroviruses. 2017;33(9):966–9. 10.1089/AID.2017.0019 28316253

[14] Wandeler G, Musukuma K, Zurcher S, Vinikoor MJ, Llenas-Garcia J, Aly MM, et al Hepatitis B Infection, Viral Load and Resistance in HIV-Infected Patients in Mozambique and Zambia. PLoS One. 2016;11(3):e0152043 10.1371/journal.pone.0152043 27032097 PMC4816321

[15] Warner N, Locarnini S. Mechanisms of hepatitis B virus resistance development. Intervirology. 2014;57(3–4):218–24. 10.1159/000360940 25034491

[16] Caligiuri P, Cerruti R, Icardi G, Bruzzone B. Overview of hepatitis B virus mutations and their implications in the management of infection. World J Gastroenterol. 2016;22(1):145–54. 10.3748/wjg.v22.i1.145 26755866 PMC4698481

[17] Shirvani-Dastgerdi E, Winer BY, Celià-Terrassa T, Kang Y, Tabernero D, Yagmur E, et al Selection of the highly replicative and partially multidrug resistant rtS78T HBV polymerase mutation during TDF-ETV combination therapy. J Hepatol. 2017;67(2):246–54. 10.1016/j.jhep.2017.03.027 28392234 PMC6016549

[18] Cooreman MP, Leroux-Roels G, Paulij WP. Vaccine- and hepatitis B immune globulin-induced escape mutations of hepatitis B virus surface antigen. J Biomed Sci. 2001;8(3):237–47. 10.1159/000054039 11385295

[19] Fischer KP, Gutfreund KS, Tyrrell DL. Lamivudine resistance in hepatitis B: mechanisms and clinical implications. Drug Resist Updat. 2001;4(2):118–28. 10.1054/drup.2001.0190 11512520

[20] Aoudjane S, Chaponda M, Gonzalez Del Castillo AA, O’Connor J, Noguera M, Beloukas A, et al Hepatitis B virus sub-genotype A1 infection is characterized by high replication levels and rapid emergence of drug resistance in HIV-positive adults receiving first-line antiretroviral therapy in Malawi. Clin Infect Dis. 2014 12;59(11):1618–26. 10.1093/cid/ciu630 25100867 PMC4650769

[21] Sheldon J, Camino N, Rodés B, Bartholomeusz A, Kuiper M, Tacke F, et al Selection of hepatitis B virus polymerase mutations in HIV-coinfected patients treated with tenofovir. Antivir Ther. 2005;10(6):727–34. 16218172

[22] Zoulim F. Hepatitis B virus resistance to antiviral drugs: where are we going? Liver Int. 2011;31(s1):111–6. 21205147 10.1111/j.1478-3231.2010.02399.x PMC3096621

[23] Patzer RE, McClellan WM. Influence of race, ethnicity and socioeconomic status on kidney disease. Nat Rev Nephrol. 2012;8(9):533–41. 10.1038/nrneph.2012.117 22735764 PMC3950900

[24] Agbaji OO, Agaba PA, Idoko JA, Taiwo B, Murphy R, Kanki P, et al Temporal changes in renal glomerular function associated with the use of Tenofovir Disoproxil Fumarate in HIV-infected Nigerians. West Afr J Med. 2011; 30(3):164–8. 22120479

[25] Buti M, Riveiro-Barciela M, Esteban R. Tenofovir Alafenamide Fumarate: A New Tenofovir Prodrug for the Treatment of Chronic Hepatitis B Infection. J Infect Dis. 2017;216(suppl_8):S792–6. 10.1093/infdis/jix135 29156043

[26] Lee HW, Chang HY, Yang SY, Kim HJ. Viral evolutionary changes during tenofovir treatment in a chronic hepatitis B patient with sequential nucleos(t)ide therapy. J Clin Virol. 2014;60(3):313–6. 10.1016/j.jcv.2014.03.018 24836314

[27] Consolidated guidelines on the use of antiretroviral drugs for treating and preventing HIV infection: Recommendations for a public health approach—Second edition http://www.who.int/hiv/pub/arv/chapter4.pdf?ua=1

[28] World Health Organisation–South Africa HIV Country Profile: 2016 http://www.who.int/hiv/data/Country_profile_South_Africa.pdf

[29] Jooste P, van Zyl A, Adland E, Daniels S, Hattingh L, Brits A, et al Screening, characterisation and prevention of Hepatitis B virus (HBV) co-infection in HIV-positive children in South Africa. J Clin Virol. 2016;85:71–4. 10.1016/j.jcv.2016.10.017 27838494 PMC5142290

[30] McMahon MA, Jilek BL, Brennan TP, Shen L, Zhou Y, Wind-Rotolo M, et al The HBV Drug Entecavir—Effects on HIV-1 Replication and Resistance. N Engl J Med. 2007;356(25):2614–21. 10.1056/NEJMoa067710 17582071 PMC3069686

[31] Locarnini SA. The Hepatitis B Virus and Antiviral Drug Resistance: Causes, Patterns and Mechanisms. In: Antimicrobial Drug Resistance; 2017p. 565–77.

[32] Nelson NP, Jamieson DJ, Murphy T V. Prevention of Perinatal Hepatitis B Virus Transmission. J Pediatric Infect Dis Soc. 2014;3 Suppl 1:S7–12. 25232477 10.1093/jpids/piu064 PMC4164184

[33] Wilson P, Parr JB, Jhaveri R, Meshnick SR. Call to Action: Prevention of Mother-to-Child Transmission of Hepatitis B in Africa. J Infect Dis. 2018;217(8):1180–3. 10.1093/infdis/jiy028 29351639 PMC6279162

[34] WHO UNICEF coverage estimates WHO World Health Organization: Immunization, Vaccines And Biologicals. Vaccine preventable diseases Vaccines monitoring system 2017 Global Summary Reference Time Series: HEPB3. http://apps.who.int/immunization_monitoring/globalsummary/timeseries/tswucoveragehepb3.html

[35] Hsu H-Y, Chang M-H, Liaw S-H, Ni Y-H, Chen H-L. Changes of hepatitis B surface antigen variants in carrier children before and after universal vaccination in taiwan. Hepatology. 1999;30(5):1312–7. 10.1002/hep.510300511 10534356

[36] Hayer J, Jadeau F, Deléage G, Kay A, Zoulim F, Combet C. HBVdb: a knowledge database for Hepatitis B Virus.Nucleic Acids Res. 2013 41: D566–D570. 10.1093/nar/gks1022 23125365 PMC3531116

[37] Bell TG, Yousif M, Kramvis A. Bioinformatic curation and alignment of genotyped hepatitis B virus (HBV) sequence data from the GenBank public database. Springerplus. 2016 5(1):1896 10.1186/s40064-016-3312-0 27843753 PMC5084120

[38] Simmonds P. SSE: a nucleotide and amino acid sequence analysis platform. BMC Res Notes. 2012 5(1):50. 22264264 10.1186/1756-0500-5-50 PMC3292810

[39] Mokaya J, Hadley M, Matthews P. gene.alignment.tables. Figshare. 2017. 10.6084/m9.figshare.5729229

[40] Mokaya J, Hadley M, Matthews P. On-line tool to visualise sites of drug and vaccine escape mutations within the HBV genome. 2018. https://livedataoxford.shinyapps.io/1510659619-3Xkoe2NKkKJ7Drg/

[41] Hundie GB, Raj VS, Michael DG, Pas SD, Osterhaus ADME, Koopmans MP, et al Molecular epidemiology and genetic diversity of hepatitis B virus in Ethiopia. J Med Virol. 2016;88(6):1035–43. 10.1002/jmv.24437 26629781

[42] Mahgoub S, Candotti D, El Ekiaby M, Allain J-P. Hepatitis B Virus (HBV) Infection and Recombination between HBV Genotypes D and E in Asymptomatic Blood Donors from Khartoum, Sudan. J Clin Microbiol. 2011;49(1):298–306. 10.1128/JCM.00867-10 21048009 PMC3020474

[43] Amponsah-Dacosta E, Lebelo RL, Rakgole JN, Selabe SG, Gededzha MP, Mayaphi SH, et al Hepatitis B virus infection in post-vaccination South Africa: Occult HBV infection and circulating surface gene variants. J Clin Virol. 2015;63:12–7. 10.1016/j.jcv.2014.11.032 25600597 PMC4560343

[44] Stewart B, Jobarteh ML, Sarge-njie R, Alabi A, Silva T De, Peterson K, et al Emergence of HBV resistance to lamivudine (3TC) in HIV / HBV co-infected patients in The Gambia, West Africa. BMC Res Notes. 2011;4(1):561. 22195774 10.1186/1756-0500-4-561 PMC3292846

[45] Galluzzo C, Liotta G, Andreotti M, Luhanga R, Jere H, Mancinelli S, et al Emergence of lamivudine resistance hepatitis B virus mutations in pregnant women infected with HBV and HIV receiving antiretroviral prophylaxis for the prevention of mother-to-infant transmission in Malawi. J Med Virol. 2012;84(10):1553–7. 10.1002/jmv.23365 22930502

[46] Makondo E, Bell TG, Kramvis A. Genotyping and molecular characterization of hepatitis B virus from human immunodeficiency virus-infected individuals in southern Africa. PLoS One. 2012;7(9):e46345 10.1371/journal.pone.0046345 23029487 PMC3460816

[47] Yousif M, Mudawi H, Hussein W, Mukhtar M, Nemeri O, Glebe D, et al Genotyping and virological characteristics of hepatitis B virus in HIV-infected individuals in Sudan. Int J Infect Dis. 2014;29:125–32. 10.1016/j.ijid.2014.07.002 25449246

[48] Anderson M, Gaseitsiwe S, Moyo S, Wessels MJC, Mohammed T, Sebunya TK, et al Molecular characterisation of hepatitis B virus in HIV-1 subtype C infected patients in Botswana. BMC Infect Dis. 2015;15(1):335. 26268355 10.1186/s12879-015-1096-4 PMC4535680

[49] Amponsah-Dacosta E, Rakgole JN, Gededzha MP, Lukhwareni A, Blackard JT, Selabe SG, et al Evidence of susceptibility to lamivudine-based HAART and genetic stability of hepatitis B virus (HBV) in HIV co-infected patients: A South African longitudinal HBV whole genome study. Infect Genet Evol. 2016;43:232–8. 10.1016/j.meegid.2016.05.035 27245151

[50] Deressa T, Damtie D, Fonseca K, Gao S, Abate E, Alemu S, et al The burden of hepatitis B virus (HBV) infection, genotypes and drug resistance mutations in human immunodeficiency virus-positive patients in Northwest Ethiopia. PLoS One. 2017;12(12):e0190149 10.1371/journal.pone.0190149 29281718 PMC5744989

[51] Calisti G, Muhindo R, Boum Y 2nd, Wilson LA, Foster GM, Geretti AM, et al Epidemiology of HBV infection in a cohort of Ugandan HIV-infected patients and rate and pattern of lamivudine-resistant HBV infection in patients receiving antiretroviral therapy. Trans R Soc Trop Med Hyg. 2015;109(11):723–9. 10.1093/trstmh/trv077 26386408

[52] Boyd A, Moh R, Gabillard D, le Carrou J, Danel C, Anglaret X, et al Low risk of lamivudine-resistant HBV and hepatic flares in treated HIV-HBV-coinfected patients from Cote d’Ivoire. Antivir Ther. 2015;20(6):643–54. 10.3851/IMP2959 25852125

[53] Chadwick D, Ankcorn M, Sarfo F, Phillips R, Fox Z, Garcia A, et al Outcomes of starting first-line antiretroviral therapy in hepatitis B virus / HIV-coinfected patients in Ghana. 2012; 2939–42. 10.1093/jac/dks333 22915461

[54] Geretti AM, Patel M, Sarfo FS, Chadwick D, Verheyen J, Fraune M, et al Detection of highly prevalent hepatitis B virus coinfection among HIV-seropositive persons in Ghana. J Clin Microbiol. 2010;48(9):3223–30. 10.1128/JCM.02231-09 20631103 PMC2937686

[55] Ndow G, Gore ML, Shimakawa Y, Suso P, Jatta A, Tamba S, et al Hepatitis B testing and treatment in HIV patients in The Gambia—Compliance with international guidelines and clinical outcomes. PLoS One. 2017;12(6):e0179025 10.1371/journal.pone.0179025 28614401 PMC5470698

[56] Hønge BL, Jespersen S, Medina C, Té D da S, da Silva ZJ, Lewin S, et al Hepatitis B and Delta Virus Are Prevalent but Often Subclinical Co-Infections among HIV Infected Patients in Guinea-Bissau, West Africa: A Cross-Sectional Study. PLoS One. 2014;9(6):e99971 10.1371/journal.pone.0099971 24915064 PMC4051771

[57] Faleye TOC, Adewumi MO, Ifeorah IM, Omoruyi EC, Bakarey SA, Akere A, et al Detection of hepatitis B virus isolates with mutations associated with immune escape mutants among pregnant women in Ibadan, southwestern Nigeria. Springerplus. 2015;4:43 10.1186/s40064-015-0813-1 25674500 PMC4320141

[58] Gachara G, Magoro T, Mavhandu L, Lum E, Kimbi HK, Ndip RN, et al Characterization of occult hepatitis B virus infection among HIV positive patients in Cameroon. AIDS Res Ther. 2017;14(1):11 10.1186/s12981-017-0136-0 28270215 PMC5341455

[59] Kouanfack C, Aghokeng AF, Mondain A, Bourgeois A, Kenfack A, Ducos J, et al Original article Lamivudine-resistant HBV infection in HIV-positive patients receiving antiretroviral therapy in a public routine clinic in Cameroon. Antivir Ther. 2012 326:321–6. 10.3851/IMP1911 22290198

[60] Magoro T, Gachara G, Mavhandu L, Lum E, Kimbi HK, Ndip RN, et al Serologic and genotypic characterization of hepatitis B virus in HIV-1 infected patients from South West and Littoral Regions of Cameroon. Virol J. 2016;13(1):178 10.1186/s12985-016-0636-x 27769271 PMC5073451

[61] Bivigou-Mboumba B, Francois-Souquiere S, Deleplancque L, Sica J, Mouinga-Ondeme A, Amougou-Atsama M, et al Broad Range of Hepatitis B Virus (HBV) Patterns, Dual Circulation of Quasi-Subgenotype A3 and HBV/E and Heterogeneous HBV Mutations in HIV-Positive Patients in Gabon. PLoS One. 2016;11(1):e0143869 10.1371/journal.pone.0143869 26764909 PMC4713159

[62] Bivigou-Mboumba B, Amougou-Atsama M, Zoa-Assoumou S, M’boyis Kamdem H, Nzengui-Nzengui GF, Ndojyi-Mbiguino A, et al Hepatitis B infection among HIV infected individuals in Gabon: Occult hepatitis B enhances HBV DNA prevalence. PLoS One. 2018;13(1):e0190592 10.1371/journal.pone.0190592 29315352 PMC5760027

[63] Matthews PC, Beloukas A, Malik A, Carlson JM, Jooste P, Ogwu A, et al Prevalence and characteristics of hepatitis B virus (HBV) coinfection among HIV-Positive women in South Africa and Botswana. PLoS One. 2015;10(7):1–11. 10.1371/journal.pone.0134037 PMC4517770 26218239

[64] Chambal LM, Samo Gudo E, Carimo A, Corte Real R, Mabunda N, Maueia C, et al HBV infection in untreated HIV-infected adults in Maputo, Mozambique. PLoS One. 2017;12(7):e0181836 10.1371/journal.pone.0181836 28759595 PMC5536281

[65] Andersson MI, Maponga TG, Ijaz S, Barnes J, Theron GB, Meredith SA, et al The epidemiology of hepatitis B virus infection in HIV-infected and HIV-uninfected pregnant women in the Western Cape, South Africa. Vaccine. 2013; 31(47):5579–84. 10.1016/j.vaccine.2013.08.028 23973500 PMC3898695

[66] Gededzha MP, Muzeze M, Burnett RJ, Amponsah-Dacosta E, Mphahlele MJ, Selabe SG. Complete genome analysis of hepatitis B virus in human immunodeficiency virus infected and uninfected South Africans. J Med Virol. 2016;88(9):1560–6. 10.1002/jmv.24502 26890489

[67] Powell EA, Gededzha MP, Rentz M, Rakgole NJ, Selabe SG, Seleise TA, et al Mutations associated with occult hepatitis B in HIV-positive South Africans. J Med Virol. 2015;87(3):388–400. 10.1002/jmv.24057 25164924 PMC4562318

[68] Selabe SG, Lukhwareni A, Song E, Leeuw YGM, Burnett RJ, Mphahlele MJ. Mutations associated with lamivudine-resistance in therapy-naive hepatitis B virus (HBV) infected patients with and without HIV co-infection: implications for antiretroviral therapy in HBV and HIV co-infected South African patients. J Med Virol. 2007 11;79(11):1650–4. 10.1002/jmv.20974 17854040

[69] Selabe SG, Song E, Burnett RJ, Mphahlele MJ. Frequent detection of hepatitis B virus variants associated with lamivudine resistance in treated South African patients infected chronically with different HBV genotypes. J Med Virol. 2009;81(6):996–1001. 10.1002/jmv.21479 19382250

[70] Baudi I, Iijima S, Chin’ombe N, Mtapuri-Zinyowera S, Murakami S, Isogawa M, et al Molecular epidemiology of co-infection with hepatitis B virus and human immunodeficiency virus (HIV) among adult patients in Harare, Zimbabwe. J Med Virol. 2017;89(2):257–66. 10.1002/jmv.24641 27458715

[71] WHO Clinical guidelines for the management of HIV & AIDS in adults and adolescents. 2010. http://www.who.int/hiv/pub/guidelines/south_africa_art.pdf

[72] Zhang Q, Liao Y, Cai B, Li Y, Li L, Zhang J, et al Incidence of natural resistance mutations in naïve chronic hepatitis B patients: A systematic review and meta-analysis. J Gastroenterol Hepatol. 2015;30(2):252–61. 10.1111/jgh.12831 25318660

[73] Hermans LE, Svicher V, Pas SD, Salpini R, Alvarez M, Ben Ari Z, et al Combined analysis of the prevalence of drug-resistant Hepatitis B virus in antiviral therapy-experienced patients in Europe (CAPRE). J Infect Dis. 2016;213(1):39–48. 10.1093/infdis/jiv363 26136470

[74] Meng T, Shi X, Gong X, Deng H, Huang Y, Shan X, et al Analysis of the prevalence of drug-resistant hepatitis B virus in patients with antiviral therapy failure in a Chinese tertiary referral liver centre (2010–2014). J Antimicr Res. 2017 8, p.74–81 10.1016/j.jgar.2016.10.012 28017671

[75] Khudyakov Y. Coevolution and HBV drug resistance. Antivir Ther. 2010;15(3 Part B):505–15. 20516572 10.3851/IMP1515

[76] Zoulim F, Locarnini S. Hepatitis B Virus Resistance to Nucleos(t)ide Analogues. Gastroenterology. 2009;137(5):1593–1608.e2. 10.1053/j.gastro.2009.08.063 19737565

[77] WHO Surveillance of transmitted HIV drug resistance. 2013. http://www.who.int/hiv/topics/drugresistance/surveillance/en/

[78] Cohen J. New single-day pill for HIV treatment promises more bang for less buck. Science (80-). 2017.

[79] Baran B, Soyer OM, Ormeci AC, Gokturk S, Evirgen S, Bozbey HU, et al Efficacy of tenofovir in patients with Lamivudine failure is not different from that in nucleoside/nucleotide analogue-naive patients with chronic hepatitis B. Antimicrob Agents Chemother. 2013;57(4):1790–6. 10.1128/AAC.02600-12 23380725 PMC3623318

[80] Chang M-H. HBV epidemiology in Taiwan before and after universal vaccination Review Breakthrough HBV infection in vaccinated children in Taiwan: surveillance for HBV mutants. Antivir Ther. 2010;15. 10.3851/IMP1555 20516566

[81] Hudu SA, Malik YA, Niazlin MT, Harmal NS, Sekawi Z. An Overview of Hepatitis B Virus Surface Antigen Mutant in the Asia Pacific. Curr. Issues Mol. Biol. 2014;16: 69–78. 24014801

[82] Hamada-Tsutsumi S, Iio E, Watanabe T, Murakami S, Isogawa M, Iijima S, et al Validation of Cross-Genotype Neutralization by Hepatitis B Virus-Specific Monoclonal Antibodies by In Vitro and In Vivo Infection. PLoS One. 2015;10(2):e0118062 10.1371/journal.pone.0118062 25693196 PMC4333126

[83] McNaughton A, Lourenco J, Hattingh L, Adland E, Daniels S, Zyl A van, et al. Can we meet global challenges for elimination of Hepatitis B Virus infection by 2030? Vaccine-mediated immunity in a South African cohort and a model of transmission and prevention. bioRxiv. 2017;162594.

[84] Negro F. Hepatitis D virus coinfection and superinfection. Cold Spring Harb Perspect Med. 2014;4(11):a021550 10.1101/cshperspect.a021550 25368018 PMC4208707

[85] Stockdale AJ, Chaponda M, Beloukas A, Phillips RO, Matthews PC, Papadimitropoulos A, et al Prevalence of hepatitis D virus infection in sub-Saharan Africa: a systematic review and meta-analysis. Lancet Glob Heal. 2017;5(10):e992–1003. 10.1016/S2214-109X(17)30298-X PMC5599428 28911765

[86] Betz-Stablein BD, Töpfer A, Littlejohn M, Yuen L, Colledge D, Sozzi V, et al Single-Molecule Sequencing Reveals Complex Genome Variation of Hepatitis B Virus during 15 Years of Chronic Infection following Liver Transplantation. J Virol. 2016;90(16):7171–83. 10.1128/JVI.00243-16 27252524 PMC4984637

[87] Iacomi F, Vincenti D, Vairo F, Solmone M, Mariano A, Piselli P, et al Effect of HIV co-infection on mutation patterns of HBV in patients with lamivudine-resistant chronic hepatitis B. J Med Virol. 2009;81(7):1151–6. 10.1002/jmv.21505 19475624

[88] Keane E, Funk AL, Shimakawa Y. Systematic review with meta-analysis: the risk of mother-to-child transmission of hepatitis B virus infection in sub-Saharan Africa. Aliment Pharmacol Ther. 2016;44(10):1005–17. 10.1111/apt.13795 27630001

[89] Manoto SL, Lugongolo M, Govender U. Point of Care Diagnostics for HIV in Resource Limited Settings: An Overview. medicina.2018;1–14. 30344234 10.3390/medicina54010003 PMC6037236

[90] Ellman TM, Alemayehu B, Abrams EJ, Arpadi S, Howard AA, El-Sadr WM. Selecting a viral load threshold for routine monitoring in resource-limited settings: optimizing individual health and population impact. Journal of the International AIDS Society 2017; 20(S7):e25007 10.1002/jia2.25007 PMC5978659 29171192

[91] Lange B, Roberts T, Cohn J, Greenman J, Camp J, Ishizaki A, et al Diagnostic accuracy of detection and quantification of HBV-DNA and HCV-RNA using dried blood spot (DBS) samples—a systematic review and meta-analysis. BMC Infect Dis. 2017;17(Suppl 1):693 10.1186/s12879-017-2776-z 29143616 PMC5688458

[92] Zeka AN, Tasbakan S, Cavusoglu C. Evaluation of the GeneXpert MTB/RIF assay for rapid diagnosis of tuberculosis and detection of rifampin resistance in pulmonary and extrapulmonary specimens. J Clin Microbiol. 2011;49(12):4138–41. 10.1128/JCM.05434-11 21956978 PMC3232962

[93] Jia S, Wang F, Li F, Chang K, Yang S, Zhang K, et al Rapid detection of hepatitis B virus variants associated with lamivudine and adefovir resistance by multiplex ligation-dependent probe amplification combined with real-time PCR. J Clin Microbiol. 2014;52(2):460–6. 10.1128/JCM.02554-13 24478474 PMC3911337

[94] Oxford Nanopore Technologies: https://nanoporetech.com/

[95] Kramvis A, Kew MC. Epidemiology of hepatitis B virus in Africa, its genotypes and clinical associations of genotypes. J Hep Res. 2007 37 (s1): 9–19. 10.1111/j.1872-034X.2007.00098.x 17627641

[96] Mokaya J, Hadley M, Matthews P. A systematic review of Hepatitis B virus (HBV) drug and vaccine escape mutations in Africa—Supplementary data. Figshare. 2018 10.6084/m9.figshare.5774091 PMC6095632 30080852

