# Supplementary figures and images for: A systematic review of hepatitis B virus (HBV) drug and vaccine escape mutations in Africa: A call for urgent action
Source: PLoS Negl Trop Dis. 2018 Aug 6;12(8):e0006629. doi: 10.1371/journal.pntd.0006629 (PMC6095632; doi:10.1371/journal.pntd.0006629)

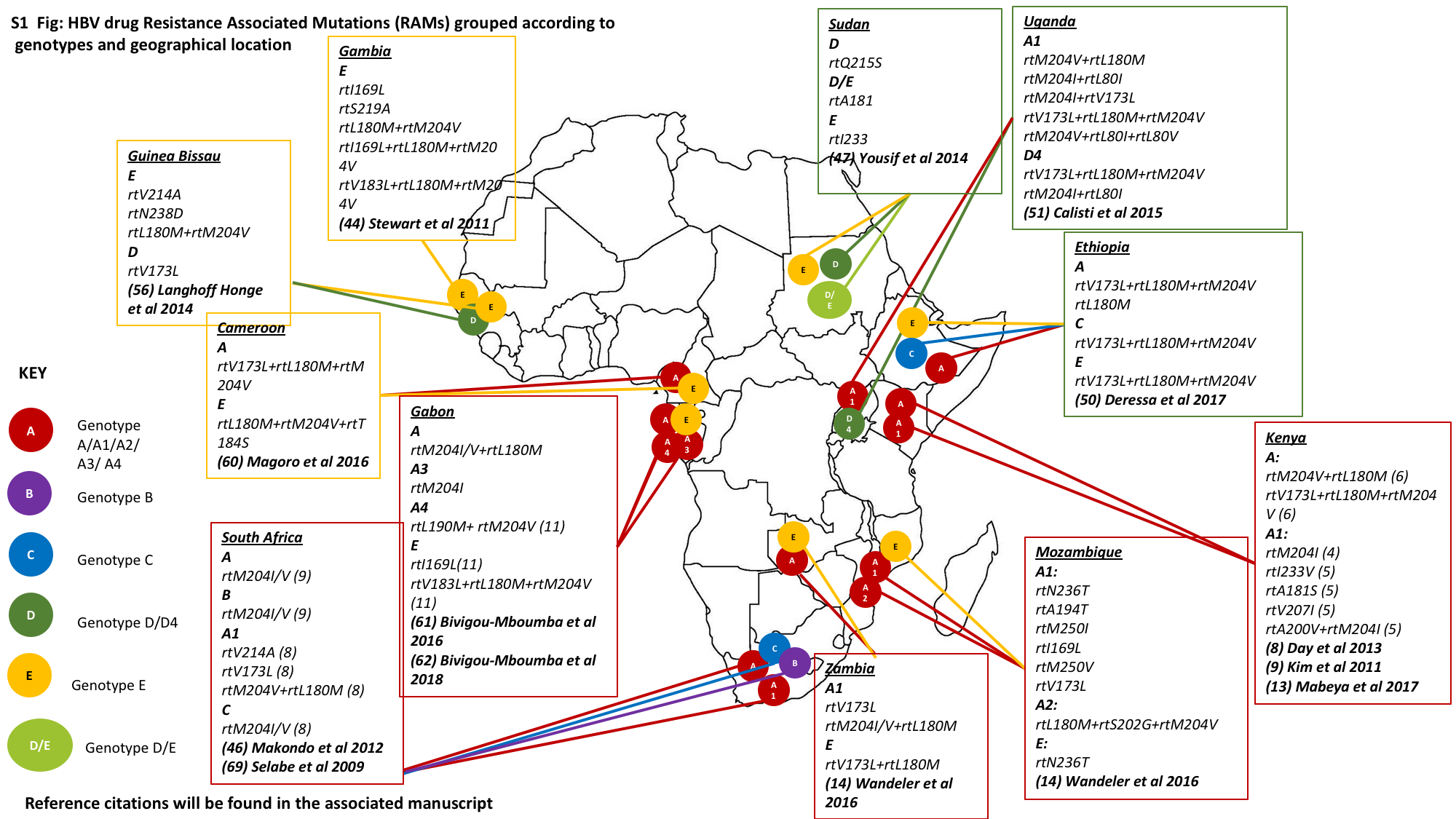

Supplement: S1 Fig — Data summarised from fourteen studies published between 2009–2017 (inclusive). 21 studies were not represented here as they did not specifically indicate which genotype individuals with RAMs belonged to. Available at https://doi.org/10.6084/m9.figshare.5774091 [96]. (TIF) [file pntd.0006629.s001.tif]

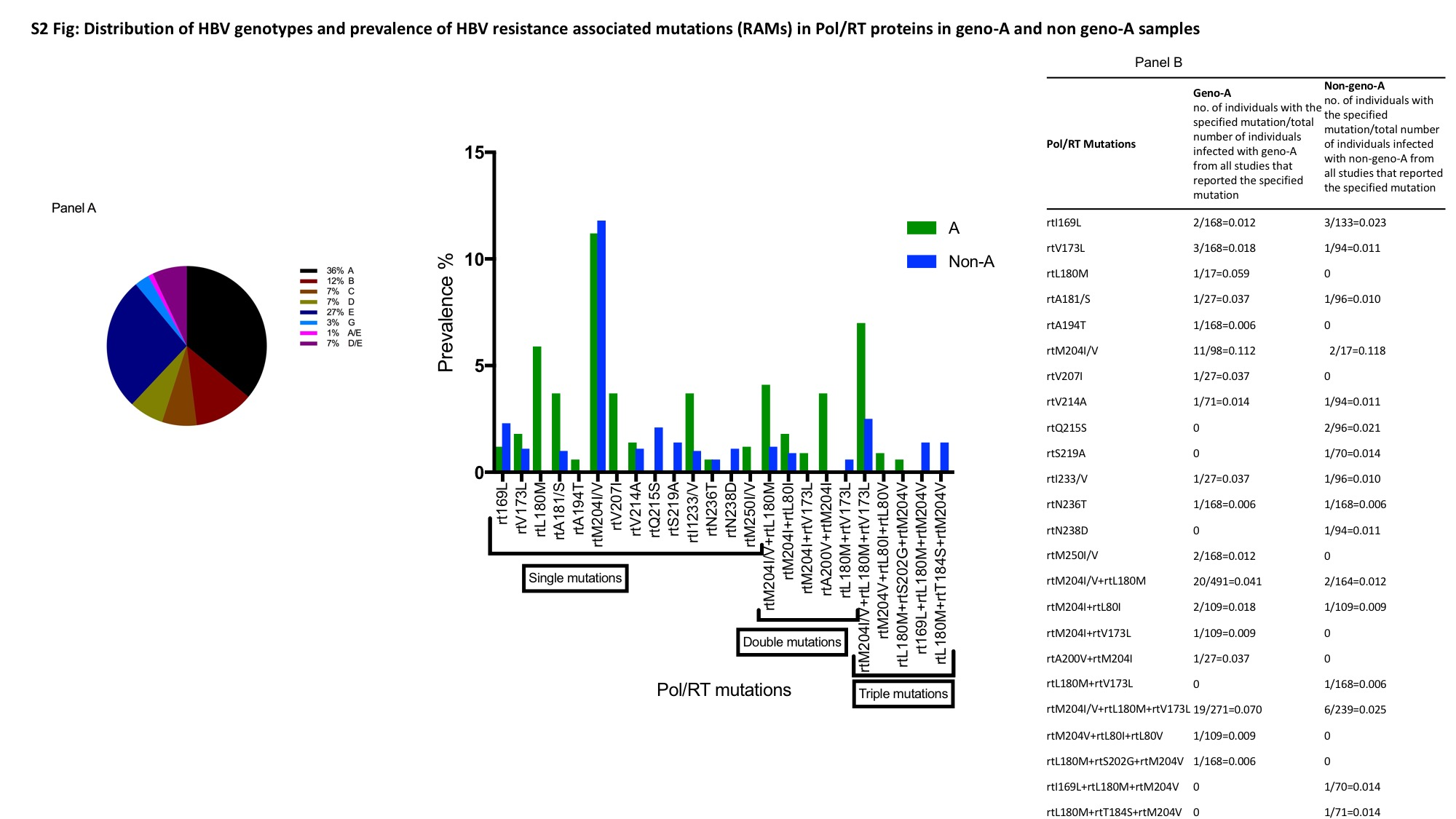

Supplement: S2 Fig — A: Distribution of HBV genotypes derived from 35 studies reporting resistance associated mutations (RAMs) in Africa published between 2009 to 2017 (inclusive); B: Prevalence of HBV resistance associated mutations (RAMs) in Pol/RT proteins in geno-A and geno-non-A samples. These data are derived from 14 studies of HBV drug resistance in Africa published between 2007 and 2017 (inclusive). 21 studies were not represented here as they did not specifically indicate which genotype individuals with RAMs belonged to. We had more geno-A samples represented than other samples, we therefore combined samples from other genotypes that had RAMs (B, C, D, E, D/E) to form geno-non-A samples. We then compared prevalence of Pol/RT mutation between geno-A samples to geno-non-A samples. Prevalence of RT/Pol mutations for a specific genotype(geno-A/geno-non-A) was determined by grouping all studies with geno-A/geno-non-A infection that reported a specific mutation; the denominator was the total number of individuals infected with geno-A/geno-non-A from these studies and the numerator was the total number of individuals infected with geno-A/geno-non-A with that specific mutation. Available at https://doi.org/10.6084/m9.figshare.5774091 [96]. (TIF) [file pntd.0006629.s002.tif]
