# Supplementary material for: A systematic review of hepatitis B virus (HBV) drug and vaccine escape mutations in Africa: A call for urgent action
Source: PLoS Negl Trop Dis. 2018 Aug 6;12(8):e0006629. doi: 10.1371/journal.pntd.0006629 (PMC6095632; doi:10.1371/journal.pntd.0006629)

PRISMA CRITERIA FOR A SYSTEMATIC REVIEW OF HEPATITIS B VIRUS (HBV) DRUG AND VACCINE ESCAPE MUTATIONS IN AFRICA: A CALL FOR URGENT ACTION

I. **PRISMA 2009 checklist**
II. **Flow diagram illustrating identification and inclusion of studies**

I. PRISMA 2009 checklist

	#	Checklist item	Section reported in manuscript
**TITLE**			
Title	1	Identify the report as a systematic review, meta-analysis, or both.	**Title**<br>A systematic review of Hepatitis B virus (HBV) drug and vaccine<br>escape mutations in Africa: a call for urgent action
**ABSTRACT**			
Structured summary	2	Provide a structured summary including, as applicable: background; objectives; data sources; study eligibility criteria, participants, and interventions; study appraisal and synthesis methods; results; limitations; conclusions and implications of key findings; systematic review registration number.	**This has been provided immediately after the title page**
**INTRODUCTION**			
Rationale	3	Describe the rationale for the review in the context of what is already known.	**Introduction section - paragraph ten**<br>To date, no systematic review has assessed the geography and prevalence of HBV RAMs and VEMs in Africa. An understanding of the extent to which these mutations circulate in Africa is essential to improving HBV therapy in patients with and without HIV coinfection. We therefore set out to describe the frequency, co-occurrence and distribution of RAMs and VEMs, and to suggest whether changes are needed in recommendations for laboratory diagnostics and/or approaches to drug therapy or vaccine deployment. This will underpin further research to identify and track relevant mutations in these populations.
Objectives	4	Provide an explicit statement of questions being addressed with reference to participants, interventions, comparisons, outcomes, and study design (PICOS).	**Introduction section - paragraph ten**<br>We therefore set out to describe the frequency,

			co-occurrence and distribution of RAMs and VEMs in Africa, and to suggest whether changes are needed in recommendations for laboratory diagnostics and/or approaches to drug therapy or vaccine deployment. This will underpin further research to identify and track relevant mutations in these populations.
**METHODS**			
Protocol and registration	5	Indicate if a review protocol exists, if and where it can be accessed (e.g., Web address), and, if available, provide registration information including registration number.	**N/A**
Eligibility criteria	6	Specify study characteristics (e.g., PICOS, length of follow-up) and report characteristics (e.g., years considered, language, publication status) used as criteria for eligibility, giving rationale.	**Methods section - paragraph one & S2 Table**<br><br>We reviewed the titles and abstracts matching the search terms and only included those relating to drug or vaccine resistance in HBV infection, including only those that presented original data and had undergone peer review. All retrieved articles were in English, therefore no exclusion in relation to language was required.<br><br>For each publication we recorded reference, publication year, study design, sample size, study population, proportion of participants who tested HBsAg+ or HBV DNA+, country, year(s) of specimen collection, genotype identified, antiviral treatment, sequencing method, gene sequenced, number of sequenced samples, participant recruitment site and sequence accession number. Data were curated using MS Excel software (Microsoft, Redmond, WA).
Information sources	7	Describe all information sources (e.g., databases with dates of coverage, contact with study authors to identify additional studies) in the search and date last searched.	**Methods section - paragraph one**<br>Between October 2017 and January 2018, we searched the published literature, in MEDLINE (PubMed; https://www.ncbi.nlm.nih.gov/pubmed), SCOPUS

			(https://www.elsevier.com/solutions/scopus) and EMBASE (https://www.elsevier.com/en-gb/solutions/embase-biomedical-research).
Search	8	Present full electronic search strategy for at least one database, including any limits used, such that it could be repeated.	**S1 Table**
Study selection	9	State the process for selecting studies (i.e., screening, eligibility, included in systematic review, and, if applicable, included in the meta-analysis).	**Methods section - paragraph one** We reviewed the titles and abstracts matching the search terms and only included those relating to drug or vaccine resistance in HBV infection, including only those that presented original data and had undergone peer review. All retrieved articles were in English, therefore no exclusion in relation to language was required.
Data collection process	10	Describe method of data extraction from reports (e.g., piloted forms, independently, in duplicate) and any processes for obtaining and confirming data from investigators.	**Methods section - paragraph two & S2 Table** For each publication we recorded reference, publication year, study design, sample size, study population, proportion of participants who tested HBsAg+ or HBV DNA+, country, year(s) of specimen collection, genotype identified, antiviral treatment, sequencing method, gene sequenced, number of sequenced samples, participant recruitment site and sequence accession number. Data were curated using MS Excel software (Microsoft, Redmond, WA).
Data items	11	List and define all variables for which data were sought (e.g., PICOS, funding sources) and any assumptions and simplifications made.	**Methods section - paragraph two & S2 Table** For each publication we recorded reference, publication year, study design, sample size, study population, proportion of participants who tested HBsAg+ or HBV DNA+, country, year(s) of specimen collection, genotype identified, antiviral treatment, sequencing method, gene sequenced, number of sequenced samples, participant recruitment site and sequence accession number. Data were curated using MS Excel software (Microsoft, Redmond, WA).

PRISMA CRITERIA FOR A SYSTEMATIC REVIEW OF HEPATITIS B VIRUS (HBV) DRUG AND VACCINE ESCAPE MUTATIONS IN AFRICA: A CALL FOR URGENT ACTION

I. **PRISMA 2009 checklist**

II. **Flow diagram illustrating identification and inclusion of studies**

Risk of bias in individual studies	12	Describe methods used for assessing risk of bias of individual studies (including specification of whether this was done at the study or outcome level), and how this information is to be used in any data synthesis.	**S2 Table**<br>This table provides detailed information of each included study. We used this information to assess for the risk of bias within and across studies. Biases identified were described in the study limitation section on page 30.
Summary measures	13	State the principal summary measures (e.g., risk ratio, difference in means).	**N/A**
Synthesis of results	14	Describe the methods of handling data and combining results of studies, if done, including measures of consistency (e.g., I^2) for each meta-analysis.	**Results section, Fig 2 & 3**<br>We described the frequency, co-occurrence and distribution of RAMs and VEMs in Africa, identified from literature and published databases

From: Moher D, Liberati A, Tetzlaff J, Altman DG, The PRISMA Group (2009). Preferred Reporting Items for Systematic Reviews and Meta-Analyses: The PRISMA Statement. PLoS Med 6(6): e1000097. doi:10.1371/journal.pmed1000097

For more information, visit: **www.prisma-statement.org**.

PRISMA CRITERIA FOR A SYSTEMATIC REVIEW OF HEPATITIS B VIRUS (HBV) DRUG AND VACCINE ESCAPE MUTATIONS IN AFRICA: A CALL FOR URGENT ACTION

I. PRISMA 2009 checklist

II. Flow diagram illustrating identification and inclusion of studies

II. Flow diagram illustrating identification and inclusion of studies

Flow diagram illustrating identification and inclusion of studies for a systematic review of drug and vaccine resistance mutations in Africa, based on PRISMA

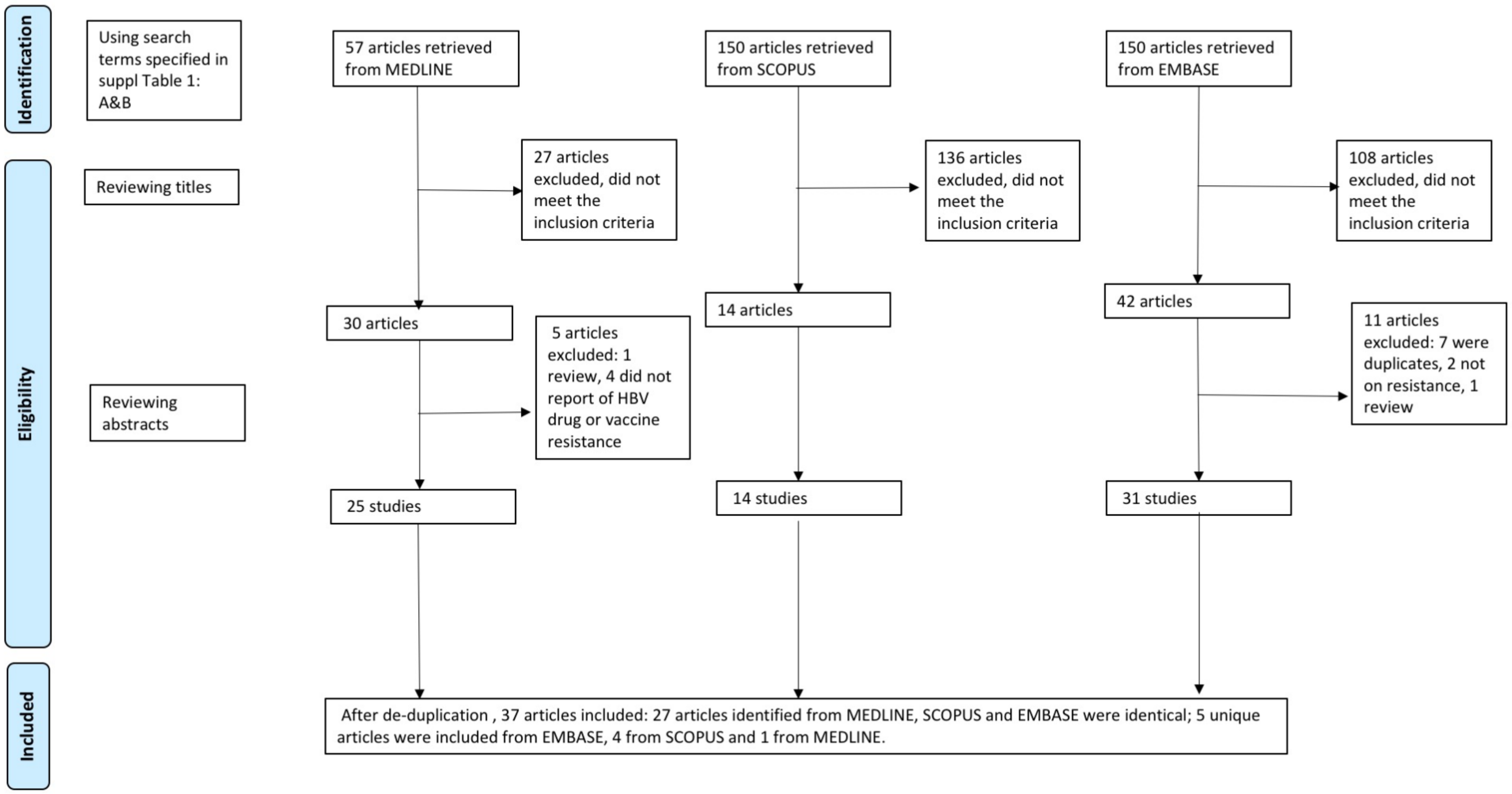

Supplement: S1 Table — Available at https://doi.org/10.6084/m9.figshare.5774091 [96]. I. Checklist to demonstrate how PRISMA criteria (2009) have been met in this review; II. Flow diagram illustrating identification and inclusion of studies for a systematic review of drug and vaccine resistance mutations in Africa. (PDF) [file pntd.0006629.s003.pdf]
