# Supplementary material for: A systematic review of hepatitis B virus (HBV) drug and vaccine escape mutations in Africa: A call for urgent action
Source: PLoS Negl Trop Dis. 2018 Aug 6;12(8):e0006629. doi: 10.1371/journal.pntd.0006629 (PMC6095632; doi:10.1371/journal.pntd.0006629)
Supplement: S2 Table — A: PubMed database; B: SCOPUS and EMBASE database. Available at https://doi.org/10.6084/m9.figshare.5774091 [96]. (PDF) [file pntd.0006629.s004.pdf]

**Suppl data Table 2A: Details of search strategy used to identify studies on HBV resistance associated mutations (RAMs) and vaccine escape mutations (VEMs) conducted in Africa, from PubMed database.**

| **Problem (#1)** | **Region (#2)** | **Intervention (#3)** | **Outcome (#4)** |
|---|---|---|---|
| Hepatitis B virus[Mesh] *OR* "hepatitis b" *OR* HBV | Africa[MeSH] *OR* africa[tiab] *OR* "sub Saharan africa"[tiab] *OR* "sub-Saharan Africa"[tiab] *OR* Angola[tiab] *OR* Benin[tiab] *OR* Botswana[tiab] *OR* "Burkina Faso"[tiab] *OR* Burundi[tiab] *OR* Cameroon[tiab] *OR* "Cape Verde"[tiab] *OR* "Central African Republic"[tiab] *OR* Chad[tiab] *OR* Comoros[tiab] *OR* "Republic of the Congo"[tiab] *OR* "Democratic Republic of the Congo"[tiab] *OR* "Cote d'Ivoire"[tiab] *OR* Djibouti[tiab] *OR* "Equatorial Guinea"[tiab] *OR* Eritrea[tiab] *OR* Ethiopia[tiab] *OR* Gabon[tiab] *OR* "The Gambia"[tiab] *OR* Ghana[tiab] *OR* Guinea[tiab] *OR* "Guinea-Bissau"[tiab] *OR* Kenya[tiab] *OR* Lesotho[tiab] *OR* Liberia[tiab] *OR* Madagascar[tiab] *OR* Malawi[tiab] *OR* Mali[tiab] *OR* Mauritania[tiab] *OR* Mauritius[tiab] *OR* Mozambique[tiab] *OR* Namibia[tiab] *OR* Niger[tiab] *OR* Nigeria[tiab] *OR* Rwanda[tiab] *OR* "Sao | antiviral* *OR* lamivudine *OR* LAM *OR* 3TC *OR* adefovir *OR* ADV *OR* telbivudine *OR* LdT *OR* entecavir *OR* ETV *OR* Tenofovir *OR* TDF | resista* *OR* drug muta* *OR* DRMs *OR* RAMs OR vaccine escap* *OR* vaccine mut* *OR* VEMs *OR* escap* |

| | | | |
|---|---|---|---|
| | Tome and Principe"[tiab] *OR* Senegal[tiab] *OR* Seychelles[tiab] *OR* "Sierra Leone"[tiab] *OR* "South Africa"[tiab] *OR* "South Sudan"[tiab] *OR* Sudan[tiab] *OR* Swaziland[tiab] *OR* Tanzania[tiab] *OR* Togo[tiab] *OR* Uganda[tiab] *OR* Zambia[tiab] *OR* Zimbabwe[tiab] | | |

*The terms in each row was combined by Boolean operator *OR,* the columns were combined by Boolean term *AND (#1 AND #2 AND #3 AND #4)*

**Suppl data Table 2B: Details of search strategy used to identify studies on HBV resistance associated mutations (RAMs) and vaccine escape mutations (VEMs) conducted in Africa, from SCOPUS and EMBASE database.**

| **Problem (#1)** | **Region (#2)** | **Intervention (#3)** | **Outcome (#4)** |
|---|---|---|---|
| "Hepatitis B virus" *OR* "hepatitis b" *OR* HBV | Africa OR "sub Saharan africa" OR Angola OR Benin OR Botswana OR "Burkina Faso" OR Burundi OR Cameroon OR "Cape Verde" OR "Central African Republic" OR Chad OR Comoros OR "Republic of the Congo" OR "Democratic Republic of the Congo" OR "Cote d'Ivoire" OR Djibouti OR "Equatorial Guinea" OR Eritrea OR Ethiopia OR Gabon OR "The Gambia" OR Ghana OR Guinea OR "Guinea-Bissau" OR Kenya OR Lesotho OR Liberia OR Madagascar OR Malawi OR Mali OR Mauritania OR Mauritius OR Mozambique OR Namibia OR Niger OR | antiviral* *OR* lamivudine *OR* LAM *OR* 3TC *OR* adefovir *OR* ADV *OR* telbivudine *OR* LdT *OR* entecavir *OR* ETV *OR* Tenofovir *OR* TDF | resista* *OR* drug muta* *OR* DRMs *OR* RAMs OR vaccine escap* *OR* vaccine mut* *OR* VEMs *OR* escap* |

| | | | |
|---|---|---|---|
| | Nigeria OR Rwanda OR "Sao Tome and Principe" OR Senegal OR Seychelles OR "Sierra Leone" OR "South Africa" OR "South Sudan" OR Sudan OR Swaziland OR Tanzania OR Togo OR Uganda OR Zambia OR Zimbabwe | | |
