# Supplementary material for: A systematic review of hepatitis B virus (HBV) drug and vaccine escape mutations in Africa: A call for urgent action
Source: PLoS Negl Trop Dis. 2018 Aug 6;12(8):e0006629. doi: 10.1371/journal.pntd.0006629 (PMC6095632; doi:10.1371/journal.pntd.0006629)
Supplement: S3 Table — Available at https://doi.org/10.6084/m9.figshare.5774091 [96]. (PDF) [file pntd.0006629.s005.pdf]

S3 Table: Full details of 37 studies identified by a systematic literature search of HBV resistance associated mutations (RAMs) and vaccine escape mutations (VEMs) from African cohorts published between 2007 and 2017 (inclusive).

Author, year & Journal	PMID	Study design	Total sample size	Characteristics of study population	HIV co-infection status of cohorta	proportion of participants who tested HBsAg + or HBV DNA+	Country	Year (s) of specimen collection	Genotypes identified	Drug treatments	Sequencing method	Gene loci sequenced	No. sequenced samples	No. of samples with detectable HBV DNA	Participant recruitment site	Accession Number
Anderson et al 2015; BMC Infect Dis.	26268355	Retrospective cross-sectional study	81	Stored plasma samples of HIV/HBV co-infected individuals collected from studies conducted in a Research Institution	+	N=81/81 (100%)	Botswana	N/A	Geno A-86%; Geno D-13%; Geno E-1%	Treatment naive	Sanger	Polymerase and Surface genes	70	Not specified	Not specified	GenBank: KR139680; KR139749

Gachara et al 2017; AIDS Res	28270215	Retrospective cross-sectional study	455	Patients attending outpatient ART health centre	+	N=20/455 (4.4%)	Cameroon	N/A	Geno A-10%; Geno E-90%	N/A	Sanger	Polymerase and overlapping surface gene (amino acids 403–768 from the EcoR1 site)	10	20	Health facility	Not specified
Kouanfack et al 2012; Antivir Ther.	22290198	Cross-sectional study	552	Patients attending outpatient ART clinic at tertiary hospitals	+	N=54/552 (9.8%)	Cameroon	2006-2007	Geno A-58%; Geno E-42%	3TC-containing ART	Sanger	Polymerase gene	12	33	Health facility	Not specified
Magoro et al 2016; Virol J.	27769271	Retrospective laboratory-based cross-sectional study	455	Patients attending outpatient ART health centre	+	N=116/455 (25.5%)	Cameroon	2013	Geno A-30%; Geno E-70%	Some patients ART naive, some were on 3TC-conta	Sanger	Polymerase and overlapping surface gene (amino acids 403–	48	48	Health facility	GenBank: KU900150-KU900195

										ining ART		768 from the EcoR1 site)				
Boyd et al 2015; Antivir Ther.	25852125	Nested cohort study	2465	Individuals enrolled in randomised multi centre trials of benefits and risks of early ART initiation	+	N=259/2465 (10.5%)	Cote d'Ivoire	2002-2011	Geno E-100%	3TC containing ART, n=82 & TDF/FTC containing ART, n=86	Sanger	Polymerase gene (amino acids 107-385) and Surface gene (amino acids 99-226)	127	127	Health facility	Not specified
Deressa et al 2017; PLoS One.	29281718	Cross-sectional study	308	Patients attending outpatient ART clinic at tertiary referral university hospital	+	N=17/308 (5.5%)	Ethiopia	2016	Geno A-41%; Geno C-6%; Geno D-12%; Geno E-12%; Geno G-6%; Undetermined-23%	3TC-containing ART, n=12; TDF + 3TC-containing ART, n=5	Sanger	Polymerase, Surface and Core genes	13	N/A	Health Facility	GenBank: pending

Hundie et al 2016; J Med Virol.	26629781	Cross sectional study	391	Stored plasma samples from HBV infected blood donors obtained from blood bank centres	±	N=391/391 (100%)	Ethiopia	2016	Geno A – 78%; Geno D – 22%	Not specified	Sanger	Surface gene (amino acids 56-808)	371	383	Community (blood donors)	GenBank: KP310929; KP311299
Bivigou-Mboumba et al 2016; PLoS One.	26764909	Cross-sectional study	762	Patients attending outpatient ART clinic	+	N=71/762 (9%)	Gabon	2010-2013	Geno A-68%; Geno E-32%	3TC-containing ART	Sanger	Polymerase, Surface and Core genes	28	114	Health facility	GenBank: KM983561-KM983588
Bivigou-Mboumba et al 2018; PLoS One.	29315352	Cross-sectional study	487	All HIV patients attending HIV care centers during study period	+	N=43/487 (8.8%)	Gabon	2018	Geno A – 60%; Geno E – 40%	3TC-containing ART, 24%; TDF -containing ART, 70%; Not specif	Sanger	Surface gene	10	43	Health Facility	GenBank: KY271377; KY271392

										ied, 24%						
Ndow et al et al 2017; PLoS One.	28614401	Cross-sectional study	870	Individuals attending HIV clinic	+	N=94/870 (10.8%)	Gambia	2015-2016	Geno A-1%; Geno E-99%	some were on ART: 3TC-containing ART n=54; TDF-containing ART, n=7	Not specified	Polymerase gene	12	52	Health facility	Not specified
Stewart et al 2011; BMC Res Notes.	22195774	Retrospective longitudinal study	570	Individuals receiving HAART; recruitment site not specified	+	N=70/570 (12.3%)	Gambia	N/A	Geno E-100%	3TC-containing ART	Sanger	Polymerase gene	21	21	Not specified	X75664; AM410963
Archampong et al 2017; Antivir Ther.	27167598	Cross-sectional study	235	Serum samples from HBV-HIV co-infected	+	N=235/235 (100%)	Ghana	2012-2014	Geno A-6%; Geno D-2%; Geno E-92%	Some were treatment naïve; some	Not specified	Polymerase gene (reverse transc	63	101	Health facility	GenBank: KU711604-KU711666

				patients collected at tertiary referral university hospital						on 3TC, n=27; TDF containing ART , n=2		riptase region)				
Chadwick et al 2012; J Antimicrob Chemother.	22915461	Retrospective longitudinal study	551	Stored sera from all adult patients attending the HIV clinic at a tertiary referral university hospital	+	N=143 /551 (26%)	Ghana	2007	N/A	3TC-containing ART	Sanger	Polymerase gene (reverse transcriptase region)	53	55	Health facility	Not specified
Geretti et al 2010; J Clin Microbiol.	20631103	laboratory-based, descriptive, cross sectional study.	838	Paired serum and plasma samples collected from HIV-infected patients attending a	+	N=140 /838 (16.7 %)	Ghana	N/A	Geno A-5%; Geno E-95%	Some ART status not known (2/3), some were on 3TC-conta	Sanger	Polymerase gene (reverse transcriptase region) and Surface gene (amin	86	118	Health facility	Not specified

				tertiary referral university hospital						ining ART (1/3)		o acids 1 to 226)				
Hønge et al 2014; PLoS One.	24915064	Cross-sectional study	576	Patients attending outpatient ART clinic at tertiary referral university hospital	+	N=94/576 (16.3%)	Guinea-Bissau	2011	Geno E-99%; Geno D-1%	some were on 3TC-containing ART	Sanger	Polymerase gene	22	42	Health facility	Not specified
Day et al 2013; PLoS One.	23527168	Prospective cohort study	159	Longitudinal cohort study of female sex workers in an urban setting	+	N=11/159 (6.9%)	Kenya	N/A	Geno A-100%	3TC-containing ART	Not specified	Polymerase gene (YMDD region)	10	10	Community (Female sex workers)	Not specified
Kim et al 2011; J Viral Hepat.	21914062	Prospective longitudinal study	389	Individuals from an urban centre enrolled in randomis	+	N=27/389 (6.9%)	Kenya	2006-2008	Geno A-100%	3TC-containing ART	Sanger	Polymerase gene	19	21	Health facility	Not specified

				ed controlled trial of adherence to ART												
Mabeya et al 2017; AIDS Res Hum Retroviruses.	28316253	Cross-sectional study	400	Individuals seeking treatment at the comprehensive HIV Clinic at tertiary referral university hospital	+	N=29/ 400 (7.3%)	Kenya	2015	Geno A-100%	some on 3TC-containing ART, n= 55%; TDF-containing ART, n= 45%	Sanger	Polymerase gene (reverse transcriptase region)	11	11	Health facility	Not specified
Aoudjane et al 2014; Clin Infect Dis.	25100867	Prospective longitudinal study	1117	Individuals starting ART treatment at a tertiary referral university hospital	+	N=133 /1117 (11.9 %)	Malawi	2007 - 2009	Geno A-99%; Geno E-1%	3TC-containing ART	Sanger and Deep sequencing by Illumina	Polymerase gene (reverse transcriptase region)	120	133	Health facility	Not specified

Galluzzo et al 2012; J Med Virol.	22930502	Prospective longitudinal study	21	Pregnant women enrolled in a PMTCT study on safety and pharmacokinetics of antiretroviral drugs	+	N=21/21 (100%	Malawi	2008-2009	Geno A-99%; Geno E-1%	3TC-containing ART	Not specified	Polymerase and surface genes	12	12	Not specified	Not specified
Chambal et al 2017; PLoS One.	29267379	Cross-sectional study	518	Patients attending outpatient ART health centre	+	N=47/518 (9.1%)	Mozambique	2012	Geno A-93%; Geno E-7%	Treatment naive	Sanger	Surface genes and overlapping polymerase (reverse transcriptase region)	27	46	Health facility	Not specified
Wandeler et al 2016;	27032097	Prospective cross-section	1032	Individuals starting ART treatmen	+	N=168/1032 (16.3%)	Mozambique and Zambia	2013-2014	Geno A-59%; Geno E-38%;	Treatment naive	Sanger	Polymerase gene (amino acids	102	156	Health facility	Not specified

PLoS One.		al study		t at urban clinic in Mozambique and rural clinic in Zambia					Geno A/E-1%			18–330)				
Faleye et al 2015; Springerplus.	25674500	Cross-sectional study	272	Pregnant women attending antenatal clinics from two tertiary university hospitals	±	N=15/272 (5.5%)	Nigeria	2012-2013	Geno E-71%	Not specified	Not specified	Surface genes	7	15	Health Facility	GenBank: KM225621-KM225627
Amponsah-Dacosta et al 2015; J Clin Virol	25600597	Cross-sectional study	201	Stored serum of individuals exposed to HBV participating in a health facility-based hepatitis B serosurv	±	N=33/201 (16.4%)	South Africa	N/A	Geno A1-92%; Geno A2- 5%; Geno D4- 3%	Not specified	Not specified	Surface genes	37	149	Not specified	Not specified

				ey conducted at a provincial level.												
Amponsah-Dacosta et al 2016; Infect Genet Evol.	27245151	Retrospective longitudinal study	5	Individuals due to HAART initiation enrolled in longitudinal study	+	N=5/5 (100%)	South Africa	N/A	Geno A-100%	3TC-containing ART, n=4	Sanger	Complete genome	4	5	Not specified	Not specified
Andersson et al 2013; Vaccine.	23973500	Retrospective cross-sectional study	3099	Stored serum of women infected with HIV enrolled in an Antenatal Sentinel HIV and Syphilis Prevalence Survey	±	N=97/3099 (3.1%)	South Africa	2008	Geno A-93%; Geno D-7%	Some were on 3TC-containing ART; some on TDF-containing ART	Not specified	Surface genes	68	78	Health facility	Not specified
Gededzha et al 2016; J Med Virol.	26890489	Laboratory based cross-section	9	Stored sera from HBV infected individua	±	N=9/9 (100%)	South Africa	2007-2011	Geno A-56%; Geno C-22%; Geno D-22%	some were on 3TC-conta	Sanger	Complete genome	9	9	Health facility	GenBank: KT347087-KT347092; GQ184323; GQ184326; GQ167301

		al study		ls attending a tertiary referral university hospital						ining ART						
Makondo et al 2012; PLoS One.	23029487	Laboratory-based descriptive cross-sectional study.	298	Stored sera from HIV infected individuals prior to ART initiation, recruitment site not specified	+	N=71/298 (23.8%)	South Africa	N/A	Geno A-99%; Geno D-1%	Treatment naive	Sanger	Surface and Core genes	71	71	Not specified	GenBank/EMBL/DDBJ: JX144270-JX144323
Powell et al 2015; J Med Virol	25164924	Cross-sectional study	394	Stored serum samples of individuals infected with HIV receiving care at a tertiary universit	+	N=37/394 (9.4%)	South Africa	2004-2009	Geno A1-24%; Geno A2-69%; Geno D4- 6%	Not specified	Not specified	Polymerase and Surface genes	49	90	Health facility	GenBank: KF475982; KF476030

				y hospital												
Selabe et al 2007; J Med Virol.	17854040	Exploratory study	35	Individuals infected with HBV admitted at tertiary University hospital	±	N=35/35 (100%)	South Africa	N/A	N/A	Treatment naive	Sanger	Polymerase gene (YMDD region)	35	35	Health facility	GenBank: DQ529242-DQ529244
Selabe et al 2009; J Med Virol.	19382250	Retrospective cross-sectional study	17	Individuals infected with HBV admitted at tertiary University hospital	-	N=17/17 (100%)	South Africa	N/A	Geno A-59%; Geno B-23%; Geno C-12%; Geno E-6%	3TC-containing ART	Sanger	Polymerase, Surface and Core genes	17	17	Health facility	Not specified
Matthews et al 2015; PLoS One.	26218239	Retrospective cross-sectional study	1022	Women attending antenatal and paediatric clinics	±	N=72/1022 (7%)	South Africa & Botswana	2015	Geno A-88%; Geno D-12%	Treatment naive	Sanger	Polymerase gene (reverse transcriptase region)	16	30	Health facility	Not specified

Hamers et al 2013; J Acquir Immune Defic Syndr.	23892239	Multicentre prospective cohort study	1087	Individuals enrolled in a multicentre prospective study of ART resistance monitoring	+	N=92/ 1087 (8.5%)	South Africa & Zambia	2007 - 2008	Geno A-76%; Geno D-2%; Geno E-22%	3TC-containing ART, n=54; TDF-containing ART, n=48	Not specified	Polymerase gene (including major part of S and part of pre-S2 gene)	54	70	Not specified	Not specified
Yousif et al 2014; Int J Infect Dis.	25449246	Cross-sectional study	358	Individuals seeking treatment at a AIDS care unit and HIV treatment centre	+	N=96/ 358 (26.8 %)	Sudan	N/A	Geno A-19%; Geno D-46%; Geno E-22%; Geno D/E-14%	Treatment naive	Not specified	Polymerase, Surface and Core genes	46	96	Not specified	GenBank: KM108588; KM108626
Mahgoub et al 2011; J Clin Microbiol.	21048009	Cross-sectional study	404	Plasma samples from blood donors from capital city in Sudan	±	N=16/ 404 (4%)	Sudan	2008	Geno A2-2%; Geno D-41%; Geno E-56%	Not specified	Not specified	Surface and Core genes	S-47, BCP/ PC-22	59	Community (blood donors)	GenBank: HQ 385227

Calisti et al 2015; Trans R Soc Trop Med Hyg.	26386408	Cross-sectional study	2820	All HIV patients attending a regional referral hospital	+	N=109/2820 (3.9%)	Uganda	2009-2011	Geno A-83%; Geno D-17%	3TC-containing ART, 96%; TDF +3TC-containing ART, 4%	Sanger	Polymerase gene (reverse transcriptase region) and Surface gene (amino acids 1-226)	23	30	Health facility	Not specified
Baudi et al 2017; J Med Virol.	27458715	Laboratory-based descriptive cross-sectional study.	176	Stored plasma samples of individuals attending HIV support clinic	+	N=19/176 (10.8%)	Zimbabwe	2014	Geno A-100%	Treatment naive	Sanger	Surface gene (amino acids 20–900) and Core gene (amino acids 1,611–2,061)	7	12	Health facility	GenBank: KX648543; KX648713
