# Supplementary material for: A systematic review of hepatitis B virus (HBV) drug and vaccine escape mutations in Africa: A call for urgent action
Source: PLoS Negl Trop Dis. 2018 Aug 6;12(8):e0006629. doi: 10.1371/journal.pntd.0006629 (PMC6095632; doi:10.1371/journal.pntd.0006629)
Supplement: S4 Table — Available at https://doi.org/10.6084/m9.figshare.5774091 [96]. (PDF) [file pntd.0006629.s006.pdf]

S4 Table: HBV Pol/RT mutations among treatment naïve HBV infected patients in Africa from 12 studies published between 2007 and 2017.

Pol/RT mutation(s)	Country and number of HBV positive study participants	Author, year & Journal	HIV co-infection status of cohort[a]	Number (%) of individuals with mutation
rtI169L	South Africa & Zambia (n=92)	Hamers et al 2013; J Acquir Immune Defic Syndr.	+	1/92 (1.1%)
	Mozambique (n=168)	Wandeler et al 2016; PLoS One.	+	1/168 (0.6%)
rtV173L	Mozambique (n=168)	Wandeler et al 2016; PLoS One.	+	1/168 (0.6%)
	South Africa (n=71)	Makondo et al 2012; PLoS One.	+	1/71 (1.4%)
	Zambia (n=168)	Wandeler et al 2016; PLoS One.	+	1/168 (0.6%)
	Guinea-Bissau (n=94)	Hønge et al 2014; PLoS One.	+	1/94 (1.1%)
rtA181/S	Kenya (n=27)	Kim et al 2011; J Viral Hepat.	+	1/27 (3.7%)
	Sudan (n=96)	Yousif et al 2014; Int J Infect Dis.	+	1/96 (1.0%)
rtT184S	South Africa & Zambia (n=92)	Hamers et al 2013; J Acquir Immune Defic Syndr.	+	1/92 (1.1%)

rtA194T	Mozambique & Zambia (n=168)	Wandeler et al 2016; PLoS One.	+	1/168 (0.6%)
rtM204I	Malawi (n=47)	Galluzzo et al 2012; J Med Virol.	+	1/47 (2.1%)
	South Africa (n=37)	Powell et al 2015; J Med Virol	+	3/37 (8.1%)
	South Africa (n=35)	Selabe et al 2007; J Med Virol.	±	13/35 (37.1%)
	South Africa & Botswana (n=72)	Matthews et al 2015; PLoS One.	±	1/72 (1.4%)
rtM250I/V	Mozambique & Zambia (n=168)	Wandeler et al 2016; PLoS One.	+	1/168 (0.6%)
	Mozambique & Zambia (n=168)	Wandeler et al 2016; PLoS One.	+	1/168 (0.6%)
rtV207I	Kenya (n=27)	Kim et al 2011; J Viral Hepat.	+	1/27 (3.7%)
rtV214A	South Africa (n=71)	Makondo et al 2012; PLoS One.	+	1/71 (1.4%)
	Guinea-Bissau (n=94)	Hønge et al 2014; PLoS One.	+	1/94 (1.1%)
rtQ215S	Sudan (n=96)	Yousif et al 2014; Int J Infect Dis.	+	2/96 (2.1%)
rtI233/V	Kenya (n=27)	Kim et al 2011; J Viral Hepat.	+	1/27 (3.7%)
	Sudan (n=96)	Yousif et al 2014; Int J Infect Dis.	+	1/96 (1.0%)
rtN236T	Mozambique & Zambia (n=168)	Wandeler et al 2016; PLoS One.	+	2/168 (1.2%)

rtN238D	Guinea-Bissau (n=94)	Hønge et al 2014; PLoS One.	+	1/94 (1.1%)
rtL180M+rtM204V	Mozambique & Zambia (n=168)	Wandeler et al 2016; PLoS One.	+	1/168 (0.6%)
	South Africa (n=71)	Makondo et al 2012; PLoS One.	+	1/71 (1.4%)
	Gabon (n=71)	Bivigou-Mboumba et al 2016; PLoS One.	+	3/71 (4.2%)
rtV173L+rtL180M	Mozambique & Zambia (n=168)	Wandeler et al 2016; PLoS One.	+	1/168 (0.6%)
rtV173L+rtL180M+rtM204V	Cameroon (n=116)	Magoro et al 2016; Virol J.	+	2/116 (1.7%)
rtL180M+rtT184S+rtM204V	Cameroon (n=116)	Magoro et al 2016; Virol J.	+	1/116 (0.9%)
rtL180M+rtS202G+rtM204V	Mozambique & Zambia (n=168)	Wandeler et al 2016; PLoS One.	+	1/168 (0.6%)

* We have reported mutations within a specified HBV gene as in the index studies. However, it is possible that mutations in the polymerase gene could influence the sequence of more than one protein due to the overlapping reading frames (ORFs) in the HBV genome.

[a] HIV status is designated '+' whole cohort HIV-positive; '±' some of cohort HIV-positive; '-'none of cohort HIV-positive
