# Supplementary material for: A systematic review of hepatitis B virus (HBV) drug and vaccine escape mutations in Africa: A call for urgent action
Source: PLoS Negl Trop Dis. 2018 Aug 6;12(8):e0006629. doi: 10.1371/journal.pntd.0006629 (PMC6095632; doi:10.1371/journal.pntd.0006629)
Supplement: S5 Table — Available at https://doi.org/10.6084/m9.figshare.5774091 [96]. (PDF) [file pntd.0006629.s007.pdf]

S5 Table: Number of samples with specific Pol/RT mutations among treatment experienced HBV infected patients in Africa, from 25 studies published between 2009 and 2017 (inclusive).

Pol/RT mutation	Country	Author, year & Journal	Characteristics of study participants	Number (%) of individuals with mutation
rtI169L	Gambia (n=70)	Stewart et al 2011; BMC Res Notes.	+	1/70 (1.4%)
	Gabon (n=43)	Bivigou-Mboumba et al 2018; PLoS One.	+	1/43 (2.3%)
	Ethiopia (n=391) #	Hundie et al 2016; J Med Virol.	±	1/391 (0.3%)
rtV173L	South Africa (n=97)	Andersson et al 2013; Vaccine.	±	1/97 (1.0%)
rtL180M	Ethiopia (n=17)	Deressa et al 2017; PLoS One.	+	1/17 (6%)
	Ghana (n=235)	Archampong et al 2017; Antivir Ther.	+	1/235 (0.4%)
T184A	Ethiopia (n=391) #	Hundie et al 2016; J Med Virol.	±	1/391 (0.3%)
A194T	Ethiopia (n=391) #	Hundie et al 2016; J Med Virol.	±	1/391 (0.3%)
rtM204I/V	Kenya (n=11)	Day et al 2013; PLoS One.	+	1/11 (9.1%)
	Kenya (n=27)	Kim et al 2011; J Viral Hepat.	+	1/27 (3.7%)
	Malawi (n=133)	Aoudjane et al 2014; Clin Infect Dis.	+	8/133 (6.0%)

	Malawi (n=21)	Galluzzo et al 2012; J Med Virol.	+	5/21 (23.8%)
	Gabon (n=43)	Bivigou-Mboumba et al 2018; PLoS One.	+	1/ 43 (2.3%)
	Gambia (n=94)	Ndow et al et al 2017; PLoS One.	+	2/94 (2.1%)
	South Africa (n=17)	Selabe et al 2009; J Med Virol.	-	13/17 (76.4%)
	Zambia & South Africa (n=92)	Hamers et al 2013; J Acquir Immune Defic Syndr.	+	3/92 (3.3%)
rtS219A	Gambia (n=70)	Stewart et al 2011; BMC Res Notes.	+	1/70 (1.4%)
rtN236T	Zambia & South Africa (n=92)	Hamers et al 2013; J Acquir Immune Defic Syndr.	+	1/92 (1.1%)
rtM204V/I+rtL180M	Ethiopia (n=94)	Hønge et al 2014; PLoS One.	+	1/94 (1.1%)
	Gabon (n=43)	Bivigou-Mboumba et al 2018; PLoS One.	+	1/43 (2.3%)
	Gabon (n=71)	Bivigou-Mboumba et al 2016; PLoS One.	+	9/71 (12.7%)
	Gambia (n=94)	Ndow et al et al 2017; PLoS One.	+	5/94 (5.3%)
	Malawi (n=21)	Galluzzo et al 2012; J Med Virol.	+	1/21 (4.8%)
rtM204/IV+rtL80I	Malawi (n=133)	Aoudjane et al 2014; Clin Infect Dis.	+	1/133 (0.8%)

	Ghana (n=235)	Archampong et al 2017; Antivir Ther.	+	1/235 (0.4%)
	Uganda (n=109)	Calisti et al 2015; Trans R Soc Trop Med Hyg.	+	3/109 (2.8%)
rtA200V+rtM204I	Kenya (n=27)	Kim et al 2011; J Viral Hepat.	+	1/27 (3.7%)
rtV173L+rtA194T	Ghana (n=140)	Geretti et al 2010; J Clin Microbiol.	+	1/140 (0.7%)
rtL180M+rtM204I	Ghana (n=235)	Archampong et al 2017; Antivir Ther.	+	1/235 (0.4%)
rtM204V+rtL180M	Cameroon (n=20)	Gachara et al 2017; AIDS Res	+	1/20 (5%)
	Cameroon (n=54)	Kouanfack et al 2012; Antivir Ther.	+	5/54 (9.3%)
	Gabon (43)	Bivigou-Mboumba et al 2018; PLoS One.	+	1/43 (2.3%)
	Gambia (n=70)	Stewart et al 2011; BMC Res Notes.	+	2/70 (2.9%)
	Ghana (n=235)	Archampong et al 2017; Antivir Ther.	+	1/235 (0.4%)
	Ethiopia (n=391) #	Hundie et al 2016; J Med Virol.	±	5/391 (1.3%)
	Kenya (n=29)	Mabeya et al 2017; AIDS Res Hum Retroviruses.	+	1/29 (3.4%)
	Malawi (n=133)	Aoudjane et al 2014; Clin Infect Dis.	+	10/133 (7.5%)
	Uganda (n=109)	Calisti et al 2015; Trans R Soc Trop Med Hyg.	+	3/109 (2.8%)

	Guinea-Bissau (n=94)	Hønge et al 2014; PLoS One.	+	1/94 (1.1%)
rtM204I+rtV173L	Uganda (n=109)	Calisti et al 2015; Trans R Soc Trop Med Hyg.	+	1/109 (0.9%)
rtM204I/V+rtV173L+rtL180M	Cameroon (n=20)	Gachara et al 2017; AIDS Res	+	1/20 (5%)
	Cameroon (n=54)	Kouanfack et al 2012; Antivir Ther.	+	1/54 (1.9%)
	Cameroon (n=116)	Magoro et al 2016; Virol J.	+	4/116 (3.4%)
	Cote d'Ivoire (n=259)	Boyd et al 2015; Antivir Ther.	+	2/259 (0.8%)
	Gabon (n=43)	Bivigou-Mboumba et al 2018; PLoS One.	+	2/43 (4.7%)
	Ethiopia (n=17)	Deressa et al 2017; PLoS One.	+	6/17 (35%)
	Gambia (n=94)	Ndow et al et al 2017; PLoS One.	+	3/94 (3.2%)
	Gambia (n=70)	Stewart et al 2011; BMC Res Notes.	+	1/70 (1.4%)
	Ghana (n=140)	Geretti et al 2010; J Clin Microbiol.	+	3/140 (2.1%)
	Ghana (n=235)	Archampong et al 2017; Antivir Ther.	+	5/235 (2.1%)
	Kenya (n=29)	Mabeya et al 2017; AIDS Res Hum Retroviruses.	+	5/29 (17.2%)
	Malawi (n=133)	Aoudjane et al 2014; Clin Infect Dis.	+	4/133 (3.0%)

	Uganda (n=109)	Calisti et al 2015; Trans R Soc Trop Med Hyg.	+	5/109 (4.6%)
rtM204V+rtL180M+rtA181S	Malawi (n=133)	Aoudjane et al 2014; Clin Infect Dis.	+	2/133 (1.5%)
rtM204V+rtL80I+rtL180M	Ghana (n=235)	Archampong et al 2017; Antivir Ther.	+	2/235 (0.9%)
	Malawi (n=133)	Aoudjane et al 2014; Clin Infect Dis.	+	1/133 (0.8%)
rtM204I+rtL80I+rtL80V	Uganda (n=109)	Calisti et al 2015; Trans R Soc Trop Med Hyg.	+	1/109 (0.9%)
rtL180M+rtS202G+rtM204V	Cameroon (n=54)	Kouanfack et al 2012; Antivir Ther.	+	1/54 (1.9%)
rtL180M+rtM204I+rtT184S	Cameroon (n=20)	Gachara et al 2017; AIDS Res	+	1/20 (5%)
rtV173L+rtL180M+rtM204I	Ghana (n=235)	Archampong et al 2017; Antivir Ther.	+	1/235 (0.4%)
rtM204I/V+/-rtL80I+/-rtV173L+/-rtL180M	Ghana (n=143)	Chadwick et al 2012; J Antimicrob Chemother.	+	12/143 (8.4%)

* We have reported mutations within a specified HBV gene as in the index studies. However, it is possible that mutations in the polymerase gene could influence the sequence of more than one protein due to the overlapping reading frames (ORFs) in the HBV genome.

[a] HIV status is designated '+' whole cohort HIV-positive; '±' some of cohort HIV-positive; '-'none of cohort HIV-positive.

Study did not specify if patients were on treatment or not.
