# Supplementary material for: A systematic review of hepatitis B virus (HBV) drug and vaccine escape mutations in Africa: A call for urgent action
Source: PLoS Negl Trop Dis. 2018 Aug 6;12(8):e0006629. doi: 10.1371/journal.pntd.0006629 (PMC6095632; doi:10.1371/journal.pntd.0006629)
Supplement: S6 Table — Information derived from published ART guidelines in all cases where these are available in the public domain. This information was collated in May 2018. Available at https://doi.org/10.6084/m9.figshare.5774091 [96]. (PDF) [file pntd.0006629.s008.pdf]

S6 Table: First line ART regimen for adults in Africa, and overlap with HBV therapy. Information derived from published ART guidelines in all cases where these are available in the public domain.

Country	1st line ARV regimen	1st line ART regimen appropriate for HBV treatment	Reference
Algeria	TDF+FTC/3TC+EFV	Yes	World Health Organisation - Algeria HIV Country Profile: 2016<br><br>http://www.who.int/hiv/data/Country_profile_Algeria_v2.pdf
Angola	AZT+3TC+NVP or TDF+3TC/FTC+EFV	Yes	World Health Organisation - Angola HIV Country Profile: 2016<br><br>http://www.who.int/hiv/data/Country_profile_Angola.pdf
Benin	TDF+3TC+EFV	Yes	World Health Organisation – Benin HIV Country Profile: 2016<br><br>http://www.who.int/hiv/data/Country_profile_Angola.pdf
Botswana	TDF/FTC+DTG	Yes	World Health Organisation - Botswana HIV Country Profile: 2016<br><br>http://www.who.int/hiv/data/Country_profile_Botswana.pdf
Burkina Faso	AZT+3TC+NVP or TDF+3TC/FTC+EFV	Yes	World Health Organisation – Burkina Faso HIV Country Profile: 2016<br><br>http://www.who.int/hiv/data/Country_profile_Burkina_Faso.pdf
Burundi	AZT+3TC+NVP or TDF+3TC+EFV	Yes	World Health Organisation - Burundi HIV Country Profile: 2016<br><br>http://www.who.int/hiv/data/Country_profile_Burundi.pdf
Cameroon	AZT+3TC+NVP or TDF+3TC+EFV	Yes	World Health Organisation - Cameroon HIV Country Profile: 2016<br><br>http://www.who.int/hiv/data/Country_profile_Cameroon.pdf
Chad	AZT+3TC+NVP or TDF+FTC+EFV	Yes	World Health Organisation - Chad HIV Country Profile: 2016

			http://www.who.int/hiv/data/Country_profile_Chad.pdf
Comoros	TDF+3TC+EFV	Yes	World Health Organisation - Comoros HIV Country Profile: 2016<br><br>http://www.who.int/hiv/data/Country_profile_Comoros.pdf
Democratic Republic of Congo	AZT+3TC+NVP or TDF+FTC/3TC+EFV	Yes	World Health Organisation - Congo HIV Country Profile: 2016<br><br>http://www.who.int/hiv/data/Country_profile_Congo.pdf
Eritrea	AZT+3TC+NVP or TDF+FTC+EFV	Yes	World Health Organisation - Eritrea HIV Country Profile: 2016<br><br>http://www.who.int/hiv/data/Country_profile_Eritrea.pdf
Eswatini (Swaziland)	TDF+3TC+ETV	Yes	World Health Organisation - Swaziland HIV Country Profile: 2016<br><br>http://www.who.int/hiv/data/Country_profile_Swaziland.pdf
Ethiopia	TDF/AZT+3TC+EFV	Yes	National Guidelines for Comprehensive HIV Prevention, Care and Treatment<br><br>https://aidsfree.usaid.gov/sites/default/files/ethiopia_art_guidelines_2017.pdf
Gambia	AZT+3TC+NVP	No	World Health Organisation - Gambia HIV Country Profile: 2016<br><br>http://www.who.int/hiv/data/Country_profile_Gambia.pdf
Ghana	AZT+3TC+NVP or TDF+FTC/3TC+EFV	Yes	World Health Organisation - Ghana HIV Country Profile: 2016<br><br>http://www.who.int/hiv/data/Country_profile_Ghana.pdf
Guinea	AZT+3TC+NVP or TDF+FTC+EFV	Yes	World Health Organisation - Guinea HIV Country Profile: 2016<br><br>http://www.who.int/hiv/data/Country_profile_Guinea.pdf
Guinea-Bissau	TDF+FTC+ETV	Yes	World Health Organisation – Guinea-Bissau HIV Country Profile: 2016

			http://www.who.int/hiv/data/Country_profile_ Guinea-Bissau.pdf
Kenya	TDF+3TC+EFV	Yes	Guidelines on use of Antiretroviral Drugs for Treating and Preventing HIV infection in Kenya<br><br>https://aidsfree.usaid.gov/sites/default/files/kenya_art_2016.pdf
Lesotho	TDF+3TC+EFV	Yes	World Health Organisation – Lesotho HIV Country Profile: 2016<br><br>http://www.who.int/hiv/data/Country_profile_Lesotho.pdf
Liberia	TDF+3TC/FTC+EFV	Yes	2nd Edition National Standard Therapeutic Guidelines and Essential Medicine List Liberia 2017<br><br>http://moh.gov.lr/wp-content/uploads/2017/12/Liberia-NSTG-EML_2nd-Edition_2017.pdf
Madagascar	TDF+3TC+EFV	Yes	World Health Organisation – Madagascar HIV Country Profile: 2016<br><br>http://www.who.int/hiv/data/Country_profile_Madagascar.pdf
Malawi	TDF+3TC+EFV	Yes	World Health Organisation – Malawi HIV Country Profile: 2016<br><br>http://www.who.int/hiv/data/Country_profile_Malawi.pdf
Mozambique	TDF+3TC+EFV	Yes	World Health Organisation – Mozambique HIV Country Profile: 2016<br><br>http://www.who.int/hiv/data/Country_profile_Mozambique.pdf
Namibia	TDF+FTC/3TC+EFV	Yes	National Guidelines for Antiretroviral therapy; 5th Edition 2016<br><br>https://aidsfree.usaid.gov/sites/default/files/na_national_guidelines_art.pdf
Niger	TDF+3TC/FTC+EFV	Yes	World Health Organisation – Niger HIV Country Profile: 2016

			http://www.who.int/hiv/data/Country_profile_Niger.pdf
Nigeria	AZT+3TC+NVP or TDF+FTC/3TC+EFV	Yes	World Health Organisation – Nigeria HIV Country Profile: 2016<br><br>http://www.who.int/hiv/data/Country_profile_Nigeria.pdf
Rwanda	TDF+3TC+EFV	Yes	World Health Organisation – Rwanda HIV Country Profile: 2016<br><br>http://www.who.int/hiv/data/Country_profile_Rwanda.pdf
Senegal	TDF+3TC+EFV	Yes	World Health Organisation – Senegal HIV Country Profile: 2016<br><br>http://www.who.int/hiv/data/Country_profile_Senegal.pdf
Sierra Leone	AZT+3TC+NVP	No	World Health Organisation – Sierra Leone HIV Country Profile: 2016<br><br>http://www.who.int/hiv/data/Country_profile_Sierra_Leone.pdf
South Africa	AZT+3TC+NVP or TDF+FTC/3TC+EFV	Yes	World Health Organisation – South Africa HIV Country Profile: 2016<br><br>http://www.who.int/hiv/data/Country_profile_South_Africa.pdf
South Sudan	AZT+3TC+NVP or TDF+3TC+EFV	Yes	World Health Organisation – South Sudan HIV Country Profile: 2016<br><br>http://www.who.int/hiv/data/Country_profile_South_Sudan.pdf
Sudan	TDF+3TC+EFV	Yes	Guidelines for the use of Antiretroviral Drugs for HIV Prevention and Treatment<br><br>http://preventcrypto.org/wp-content/uploads/2015/10/sudanartguidelines20141413103428.Pdf
Tanzania	TDF+3TC+EFV	Yes	National Guidelines for the management of HIV/AIDS: The United Republic of Tanzania<br><br>https://aidsfree.usaid.gov/sites/default/files/04_11_2016.tanzania_national_guideline_for_management_hiv_and_aids_may_2015._tagged.pdf

Togo	TDF+3TC/FTC+EFV	Yes	World Health Organisation – Togo HIV Country Profile: 2016<br><br>http://www.who.int/hiv/data/Country_profile_Togo.pdf
Uganda	TDF/FTC+DTG	Yes	World Health Organisation – Uganda HIV Country Profile: 2016<br><br>http://www.who.int/hiv/data/Country_profile_Uganda.pdf
Zambia	AZT+3TC+NVP or TDF+FTC/3TC+EFV	Yes	World Health Organisation – Zambia HIV Country Profile: 2016<br><br>http://www.who.int/hiv/data/Country_profile_Zambia.pdf
Zimbabwe	AZT+3TC+NVP or TDF+3TC+EFV	Yes	World Health Organisation – Zimbabwe HIV Country Profile: 2016<br><br>http://www.who.int/hiv/data/Country_profile_Zimbabwe.pdf

We were unable to find published guidelines for the following countries: Cape Verde, Central Africa Republic, Ivory Coast, Djibouti, Egypt, Equatorial Guinea, Gabon, Libya, Mai, Mauritania, Mauritius, Morocco, São Tomé and Príncipe, Seychelles and Tunisia.
Abbreviations: AZT – Zidovudine; DTG – Dolutegravir; EFV – Efavirenz; FTC – Emitricitabine; 3TC – Lamivudine; NVP – Nevirapine; TDF – Tenofovir.
